# CRISPR-based genome editing in primary human pancreatic islet cells

**DOI:** 10.1038/s41467-021-22651-w

**Published:** 2021-04-23

**Authors:** Romina J. Bevacqua, Xiaoqing Dai, Jonathan Y. Lam, Xueying Gu, Mollie S. H. Friedlander, Krissie Tellez, Irene Miguel-Escalada, Silvia Bonàs-Guarch, Goutham Atla, Weichen Zhao, Seung Hyun Kim, Antonia A. Dominguez, Lei S. Qi, Jorge Ferrer, Patrick E. MacDonald, Seung K. Kim

**Affiliations:** 1 Department of Developmental Biology, Stanford University School of Medicine, Stanford, CA USA; 2 Alberta Diabetes Institute and Department of Pharmacology, University of Alberta, Edmonton, AB Canada; 3 Bioinformatics and Genomics Program, Centre for Genomic Regulation (CRG), The Barcelona Institute of Science and Technology (BIST), Barcelona, Spain; 4 Centro de Investigación Biomédica en Red de Diabetes y Enfermedades Metabólicas Asociadas (CIBERDEM), Barcelona, Spain; 5 Department of Bioengineering, Stanford University, Stanford, CA USA; 6 Department of Chemical and Systems Biology, Stanford University, Stanford, CA USA; 7 Chem-H, Stanford University, Stanford, CA USA; 8 Section of Genetics and Genomics, Imperial College London, London, UK; 9 Department of Medicine (Endocrinology), Stanford University School of Medicine, Stanford, CA USA; 10 Northern California JDRF Center of Excellence, Stanford University School of Medicine, Stanford, CA USA; 11 Stanford Diabetes Research Center, Stanford University School of Medicine, Stanford, CA USA

**Keywords:** CRISPR-Cas9 genome editing, Diabetes

## Abstract

Gene targeting studies in primary human islets could advance our understanding of mechanisms driving diabetes pathogenesis. Here, we demonstrate successful genome editing in primary human islets using clustered regularly interspaced short palindromic repeats (CRISPR) and CRISPR-associated protein 9 (Cas9). CRISPR-based targeting efficiently mutated protein-coding exons, resulting in acute loss of islet β-cell regulators, like the transcription factor PDX1 and the K_ATP_ channel subunit KIR6.2, accompanied by impaired β-cell regulation and function. CRISPR targeting of non-coding DNA harboring type 2 diabetes (T2D) risk variants revealed changes in *ABCC8*, *SIX2* and *SIX3* expression, and impaired β-cell function, thereby linking regulatory elements in these target genes to T2D genetic susceptibility. Advances here establish a paradigm for genetic studies in human islet cells, and reveal regulatory and genetic mechanisms linking non-coding variants to human diabetes risk.

## Abstract (web summary)

The editing of primary human islets could provide insight into diabetes pathogenesis. Here the authors use CRISPR-Cas9 to target regulatory elements associated with T2D susceptibility.

## Introduction

Pancreatic islets of Langerhans are crucial endocrine organs that regulate internal metabolic homeostasis, and diseases like diabetes mellitus have been linked to impaired function or loss of islet cells, especially insulin-secreting β-cells. Prior studies^1–7^ have unveiled the genetic origins of islet cell dysfunction or loss in all forms of diabetes, including familial, type 1 (T1D), type 2 (T2D), and type 3c diabetes. Genetic susceptibility in T2D and T1D is driven by non-coding variants enriched in active islet enhancers^5,6,8^. Thus, experimental systems for modeling and modulating regulatory variants or elements in human islet cells could define molecular determinants of diabetes susceptibility. However, gene regulation differs substantially between human islets and experimental animal or islet-like cells produced from human cell lines^9^. For example, genes encoding the transcription factors SIX2 and SIX3 are expressed in adult human β-cells, but not in rodent β-cells^10,11^, highlighting the need for genetics in primary human islets.

Site-specific gene editing^12,13^ enabled by CRISPR, and CRISPR-associated protein 9 (Cas9) or targeted gene activation^14^ (CRISPRa) mediated by nuclease-deactivated Cas9 (dCas9), could transform our understanding of genetic mechanisms governing vital tissue and organ functions and disease development. However, it remains unknown if these modern genome editing tools can be applied to native human pancreatic islet cells: CRISPR-based gene editing or control of gene expression in primary human islet cells has not previously been reported. This reflects unmet challenges to achieve efficient, durable delivery of Cas9 and guide RNA (sgRNA) to intact islets, including the sensitivity of islet cells to methods used to achieve this delivery^15^. Furthermore, CRISPR-Cas9-based DNA editing may be less efficient in quiescent cells, like primary adult human islet cells, which are post-mitotic^16–18^ and not amenable to the selection of desired gene modifications.

Recent studies have used a strategy based on the dispersion of pancreatic islets and reaggregation into primary organoids (called pseudoislets) to enable RNA knock down or misexpression^10,19–22^. Pseudoislets are able to reconstitute native intercellular interactions of islet cells in a multicellular state that recapitulates islet cyto-architecture and function^10,20–22^. However, to date, genetic approaches in human pseudoislets have not allowed direct modification of β-cell DNA, including potential regulatory elements in non-coding DNA.

Here we describe the use of CRISPR/Cas9 and CRISPR/dCas9-based enhancer activation (CRISPRa) in primary human islets. Delivery and expression of sgRNAs and Cas9 permitted efficient genome editing, resulting in targeting of protein-coding exons and non-coding regulatory elements in primary human islet cells, loss of gene expression, and impairment of cellular function, assessed in vitro and in transplanted pseudoislets. By targeting regulatory elements with sgRNAs and dCas9 linked to the activation domains VP64-p65-Rta^23^, we also induced the expression of genes from endogenous genomic sequences of human islet cells. These experiments revealed an essential function of PDX1 in primary mature human islet β-cells, as anticipated from mouse and human genetics, and established functional target genes of regulatory elements that carry T2D-associated variants. Thus, our findings demonstrate the feasibility of manipulating coding and non-coding regulatory genomic regions in primary human islets, and thereby expand the experimental repertoire for dissecting genetic mechanisms underlying human diabetes.

## Results

### CRISPR/Cas9 targeting of *PDX1* protein-coding sequence in primary human islets

**Fig. 1:**
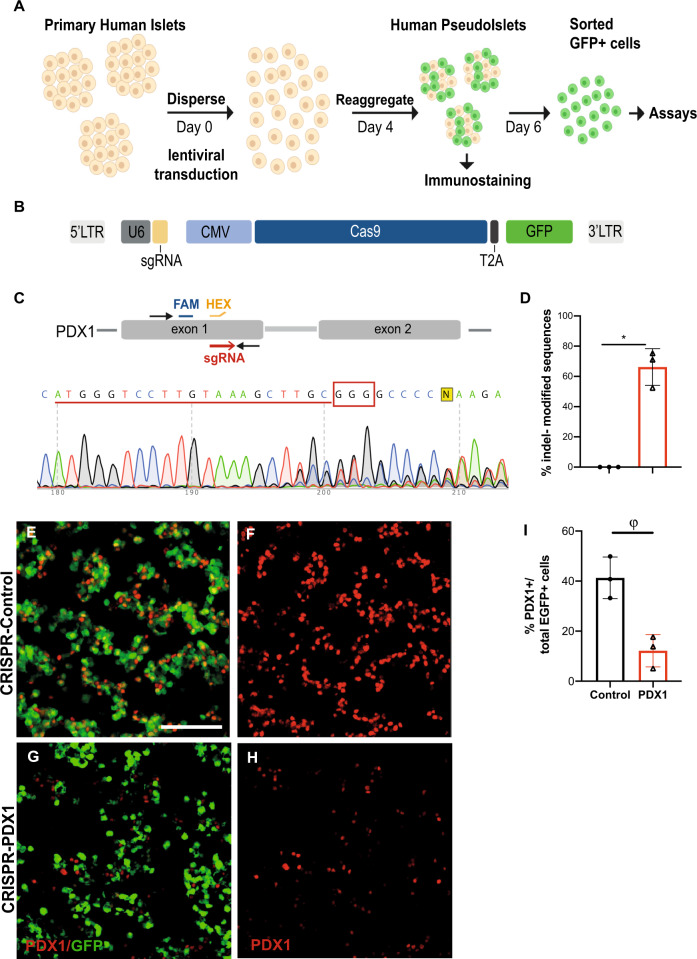
CRISPR/Cas9 targeting of *PDX1* protein-coding sequence in primary human islets. **A** Schematics of the human pseudoislet system. **B** Scheme of the lentiCRISPR construct used. **C** *PDX1* sequence, showing the sgRNA sequence (underlined in red, PAM sequence recognized by Cas9 in red box). FAM (blue) and HEX (orange) probes used for ddPCR. **D** Percentage of indel-modified sequences detected by the TIDE algorithm (*P* = 0.01). **E**–**H** Immunostaining of CRISPR-Control versus CRISPR-PDX1, showing GFP^+^ PDX1^+^ cells (*n* = 3 independent donor repetitions); scale bar: 50 μm. **I** Quantification of GFP^+^ PDX1^+^ cells for CRISPR-Control versus CRISPR-PDX1(*P* = 0.07; *n* = 3 independent donor repetitions). Data are presented as mean values ± SD. Two-tailed t tests were used to generate *P*-values. *P < 0.05, ^ϕ^*P* < 0.1. Source data are provided as a Source Data file.

**Fig. 2:**
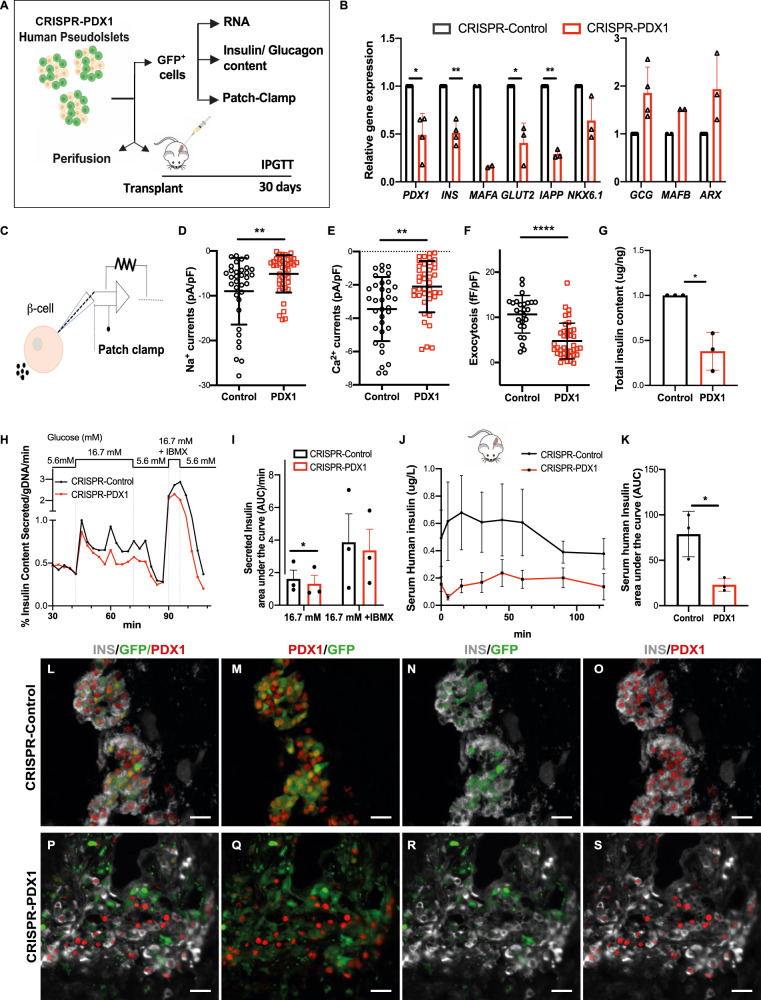
CRISPR/Cas9 targeting of *PDX1* in primary human islets leads to loss-of-function phenotypes. **A** Schematics of analyses performed to characterize the phenotype of CRISPR-PDX1 human pseudoislets. **B** Relative gene expression of known PDX1-targets in CRISPR-PDX1 GFP^+^ cells relative to CRISPR-Control GFP^+^ cells (*P* = 0.0199 for *PDX1*, *P* = 0.037 for *INS*, *P* = 0.0386 for *GLUT2*, *P* = 0.013 for *IAPP*). **C** Scheme of β-cell patch clamp. **D**–**F** β-cells show electrophysiological defects after CRISPR/Cas9 targeting of PDX1, including (**D**) impaired Na^+^ (*P* = 0.057) and (**E**) Ca^2^^+^ currents (*P* = 0.0012), and (**F**) reduced glucose-dependent β-cell exocytosis (*P* < 0.0001). **G** Total insulin content in CRISPR-PDX1 GFP^+^ cells relative to CRISPR-Control GFP^+^ cells (*P* = 0.036). **H** Secreted human insulin by CRISPR-Control versus CRISPR-PDX1 pseudoislets in vitro, following perifusion with media containing Glucose at 5.6, 6.7, and 16.7 mM +IBMX. **I** Area under the curve (AUC) of secreted human insulin by CRISPR-Control versus CRISPR-PDX1 pseudoislets at 16.7 mM (*P* = 0.0387) and 16.7 mM Glucose +IBMX. **J** Serum human insulin levels in the blood of NSG mice one-month following transplantation of CRISPR-PDX1 versus CRISPR-Control pseudoislets, following intraperitoneal glucose tolerance tests (IPGTT) (*n* = 3 independent human islets donors). **K** Area under the curve of the serum human insulin released by transplanted CRISPR-PDX1 versus CRISPR-Control pseudoislets, shown in (**J**) (*P* = 0.049). **K**–**S** Immunostaining of grafts recovered one-month following transplantation with CRISPR-Control (**K**–**O**) versus CRISPR-PDX1 (**P**–**S**) pseudoislets; INS: gray, GFP: green, PDX1: red. Scale bars: 20 um. (*n* = 3 independent donor repetitions). Data are presented as mean values ± SD for (**B**–**G**, **K**) and as mean ± SEM for (**I**) and (**J**). Two-tailed t tests were used to generate *P*-values. *P < 0.05, **P < 0.01, **** *P* < 0.0001. Source data are provided as a Source Data file.

To achieve site-specific gene editing in primary adult human islet cells, we used a strategy of islet dispersion and lentiviral transduction, followed by reaggregation and culture of islet cell clusters in pseudoislets for 6 days (Fig. 1A, Methods;^10,20^). Use of an “all-in-one” lentivirus permitted simultaneous expression of sgRNA and *Cas9* to target a protein-coding sequence in exon 1 of *PDX1*, and production of Green Fluorescent Protein from a *GFP* transgene to mark infected cells (Fig. 1B). Infection with a lenti-construct encoding a non-targeting sgRNA, *Cas9,* and *GFP* served as a control (CRISPR-Control). Islet cell transduction efficiency, determined by flow cytometry counting of GFP^+^ cells, averaged 41% (Supplementary Fig. 1A–C). We chose to target *PDX1*, which encodes a transcription factor previously shown to be crucial for β-cell function in mouse islets, and which is mutated in human diabetes^3,24–26^. After transducing PDX1 sgRNAs, we isolated GFP^+^ human islet cells 6 days after infection, and performed TIDE PCR and digital droplet (dd)PCR (Methods;^27–29^) with gene-specific probes to genomic DNA (Fig. 1C, D; *n* = 3 independent donors; Supplementary Fig. 2A, B). Insertion-deletion mutations (indels) were detected in an average of 66% of sequences by TIDE PCR; Fig. 2C, D) and in 48% of sequences by ddPCR (Supplementary Fig. 2A, B). Immunostaining studies revealed 72% reduction of GFP^+^ PDX1^+^ cells (Fig. 1E–I; *n* = 3 independent donors); thus, extensive loss of PDX1 protein in primary human islet cells corroborated our molecular findings. To assess targeting specificity, we used an in silico approach^30^ to identify the likeliest genomic sites for “off-target” modification using CRISPR/Cas9 and the sgRNA targeting *PDX1* exon 1. Sequencing analysis revealed that indels were undetectable in 7/7 predicted genomic off-target sites (Supplementary Fig. 2C, D). Thus, exposure of β-cells to CRISPR/Cas9 for several days in cultured pseudoislets achieved substantial, specific targeting in *PDX1* exon 1.

### Loss-of-function phenotypes in primary human islets from CRISPR/Cas9 targeting of *PDX1*

After CRISPR/Cas9 targeting of *PDX1*, we found that expression of β-cell-specific genes previously identified as direct targets of *Pdx1* regulation in mouse studies^26,31,32^ was also impaired in GFP^+^ human islet cells (Methods). qRT-PCR revealed a significant reduction of mRNAs encoding *PDX1* and the β-cell genes *INSULIN*, *MAFA*, *IAPP,* and *SLC2A2* (Fig. 2A, B; *n* = 4 independent donors). Total insulin content of the CRISPR-*PDX1* purified GFP^+^ cells was reduced by 60% compared to controls (Fig. 2G; *n* = 3 independent donors) and total insulin content of the CRISPR-*PDX1* intact pseudoislets was reduced by 40% (Supplementary Fig. 3A–E; *n* = 4 independent donors). We also noted an increased average expression of α-cell transcripts and glucagon content (Fig. 2B, Supplementary Fig. 3F–J; *n* = 4 independent donors), although these did not reach statistical significance. Direct PDX1 targets like *MAFA* and *SLC2A2* are regulators of β-cell function^33^, including stimulus-secretion coupling, and whole-cell patch-clamping revealed electrophysiological defects in β-cells after CRISPR/*Cas9* targeting of *PDX1*, including impaired Ca^2+^ and Na^+^ currents, and reduced glucose-stimulated β-cell exocytosis (Fig. 2C–F; *n* = 3 independent donors). In vitro perifusion assays 5 days after lentiviral infection revealed reduced glucose-dependent insulin secretion by CRISPR-PDX1 pseudoislets compared to controls (Fig. 2H–I; *n* = 3 independent donors: Methods). Together, these data revealed that CRISPR/Cas9*-*induced *PDX1* loss led to impaired physiological function in native human β-cells.

To evaluate the durability of defects and the impact of *PDX1* targeting on in vivo function, we transplanted CRISPR-PDX1 and control pseudoislets into immunocompromised, non-diabetic *NSG* mice (*n* = 3 independent donors; Methods) and assessed glucose-stimulated insulin secretion. Four weeks after transplantation, intraperitoneal glucose tolerance tests (IPGTT) revealed >70% reduction of human insulin secretion from pseudoislets with PDX1 loss compared to controls (Fig. 2J: *n* = 4 mice for CRISPR-PDX1 or for controls), and a corresponding significant reduction in human insulin output for the CRISPR-PDX1 condition (Fig. 2K: area under the curve, AUC: *P* < 0.05; Methods). Serum glucose levels after IPGTT were indistinguishable in mice transplanted with CRISPR-PDX1 or control pseudoislets (Supplementary Fig. 3K–L), likely reflecting intact host islet function. Compared to control grafts, immunostaining of grafts with *PDX1* targeting showed reduced PDX1 and INS production in GFP^+^ islet β-cells (Fig. 2P–S; Supplementary Fig. 3M, N). Together, these experiments indicate that the lentiCRISPR-pseudoislet system can be efficiently applied to primary human islet cells, providing evidence in mature human islets that *PDX1* targeting and loss leads to durable impairment of hallmark β-cell phenotypes, including cardinal physiological β-cell functions.

### CRISPR/Cas9 targeting of *KCNJ11* protein-coding sequence in primary human islets

**Fig. 3:**
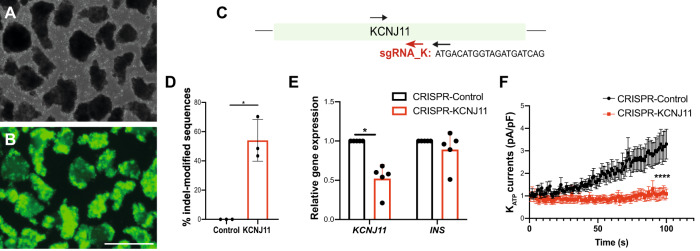
CRISPR/Cas9 targeting of *KCNJ11* protein-coding sequence in primary human islets. **A**, **B** Human pseudoislets 5 days post-infection with CRISPR-KCNJ11 lentiviruses (**A**) bright field, (**B**) blue light (488 nm), scale bar: 500 μm (*n* = 3 independent donors). **C** Schematics of KCNJ11 sequence, showing the sgRNA sequence (sgRNA_K, red). Arrows indicate primers used for PCR. **D** Percentage of indel-modified sequences detected by TIDE algorithm (*n* = 3 independent donors; *P* = 0.0225). **E** qRT-PCR of GFP^+^ cells, CRISPR-KCNJ11 (red), CRISPR-Control (Black) (*n* = 5 independent donors; *P* = 0.0175). **F** Patch clamping in single β-cells: measurement of K_ATP_ currents in CRISPR-KCNJ11 (*n* = 19 cells, 3 replicates) and CRISPR-Control (*n* = 25, 3 replicates) (*P* < 0.0001). Data are presented as mean values ± SD for (**D**–**E**) and mean values ± SEM for (**F**). Two-tailed t tests were used to generate *P*-values. *P < 0.05, **** *P* < 0.0001. Source data are provided as a Source Data file.

To assess the general use of CRISPR/Cas9 gene targeting in primary human islets, we built additional lentiCRISPR constructs to target exon 1 of *KCNJ11*, which encodes the KIR6.2 pore-forming subunit of K_ATP_ channels (Fig. 3C: Methods). Six days after transduction, we sorted GFP^+^ cells from pseudoislets (Fig. 3A, B), and used TIDE to quantify that an average 54% of sequences were indel-modified (Fig. 3D; *n* = 3 independent donors). qRT-PCR of GFP^+^ cells demonstrated a 52% *KIR6*.*2* mRNA reduction; by contrast, *INS* mRNA levels were not altered (Fig. 3E; *n* = 5 independent donors). Sequencing analysis of the likeliest genomic off-target sites (Methods;^30^) revealed that indels were undetectable in 6/6 of these sites (Supplementary Fig. 4). To assess if *KCNJ11* targeting was sufficient to impair K_ATP_ channels function, we used patch clamping in single β-cells, and confirmed that K_ATP_ currents were severely reduced after CRISPR-*KCNJ11* (Fig. 3F; *n* = 3 independent donors). Thus, CRISPR/Cas9 gene targeting can be used at distinct genetic loci to achieve loss-of-function in human β-cells.

### CRISPR/Cas9 targeting of *cis-*regulatory genomic regions in human islet cells

**Fig. 4:**
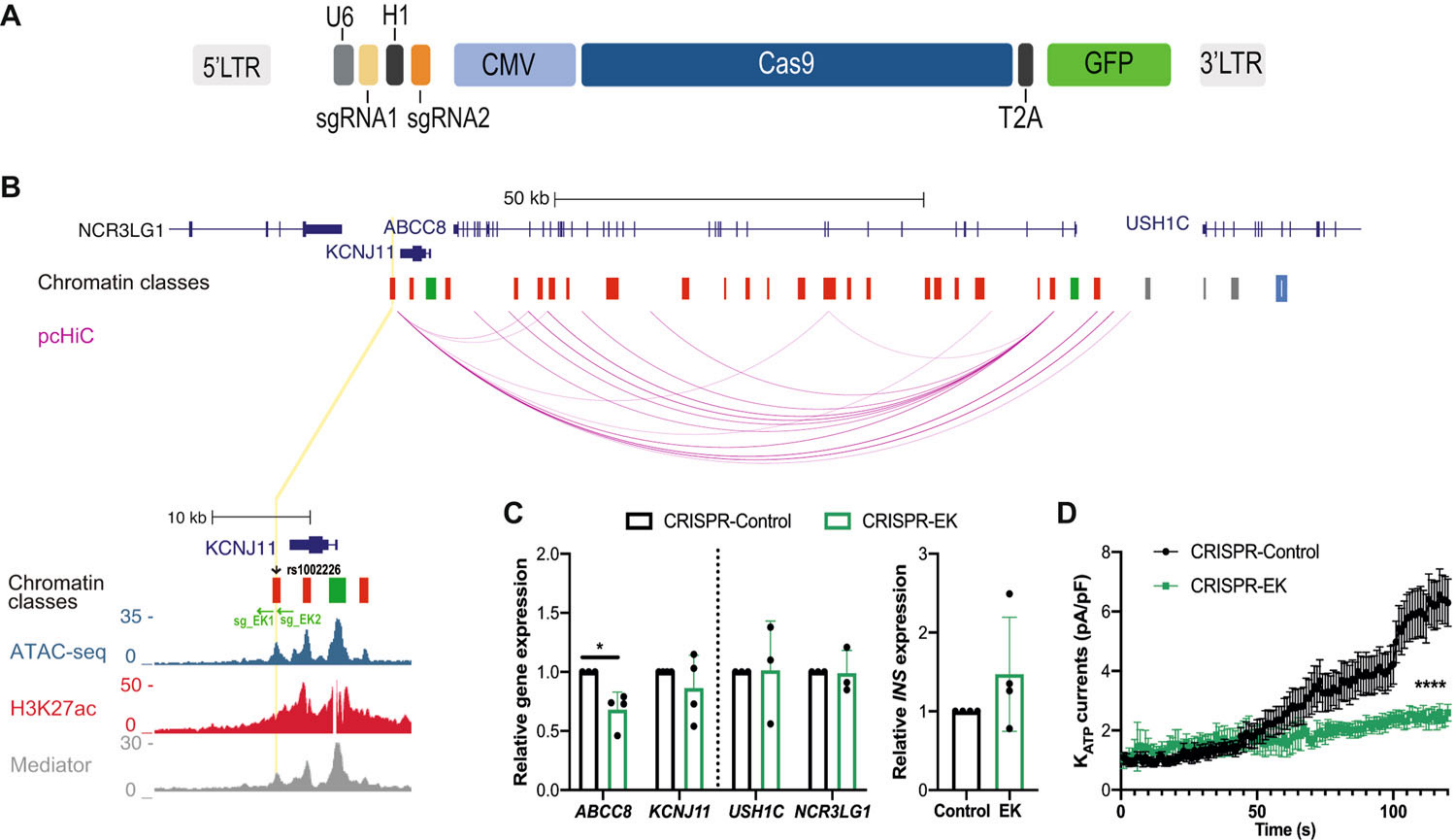
CRISPR/Cas9 targeting of a non-coding variant in the *ABCC8-KCNJ11* locus impairs *ABCC8* expression and function in primary human islets. **A** Schematic of the lenti-construct used for simultaneous expression of two sgRNAs, Cas9 and GFP (12,021 bp) in primary human islets. **B** Genome Browser tracks of the genomic context in the *KCNJ11-ABCC8* locus, arcs representing high-confidence pcHi-C interactions in human islets, highlighting variant rs1002226 (chr11:17405617) associated with diabetes risk (black arrowhead): This variant maps to a CTCF site (blue) on a class I active enhancer (yellow line, and zoomed inset); sg_EK1 and sg_EK2 flanking rs1002226 (green arrows); Chromatin classes: active promoter (green); active enhancer (red); inactive enhancer (gray); inactive open chromatin (black); strong CTCF (blue). Accessible chromatin regions in human islets are shown by ATAC-seq, H3K27ac, and Mediator ChiP-seq. **C** *ABCC8* mRNA is regulated by the rs1002226-containing enhancer in GFP^+^ pseudoislet cells, CRISPR-EK (green), CRISPR-Control (Black) (*P* = 0.0232; *n* = 4), while expression of *KCNJ11*, control genes (*USH1C*, *NCR3LG1*) in the same transcription activation domain, TAD (*n* = 3) and insulin (*n* = 4) were not modified. Data are presented as mean values +SD. **D** Measurement of K_ATP_ currents in GFP^+^ islet cells from CRISPR-EK (*n* = 26 cells, 3 replicates) and CRISPR-Control (*n* = 33, 3 replicates) (*P* < 0.0001). Data are presented as mean values ± SEM. Two-tailed t tests were used to generate *P*-values. *P < 0.05, **** *P* < 0.0001. Source data are provided as a Source Data file.

The vast preponderance of genetic risk for diabetes mellitus maps to non-coding DNA^34^, and it is known that the function of regulatory elements is extremely dependent on cell-type and on the stage of islet cell differentiation. Thus, functional studies of non-coding genomic regions in native human islets would be a major advance for islet biology and diabetes research, and we investigated if our lentiCRISPR-pseudoislet method could generate targeted deletions in postulated gene regulatory regions. The *KCNJ11-ABCC8* locus harbors non-coding variants linked to diabetes risk^1,34–38^. For example, the variant rs1002226 falls within an enhancer hub in the *KCNJ11-ABCC8* locus associated with diabetes risk, and we adapted our lentiCRISPR construct to simultaneously express two sgRNAs (sg_EK1 and sg_EK2) and induce a small deletion in the putative enhancer encompassing this risk SNP (Fig. 4A, B: Methods;^35,39^). In purified GFP^+^ cells, qRT-PCR analysis revealed a reduction of *ABCC8* mRNA, while *KCNJ11* mRNA was not significantly changed (Fig. 4C; *n* = 4 independent donors). This finding corresponds well with data from our prior chromatin looping pc-HiC studies^35^ revealing an interaction linking the putative enhancer region harboring rs1002226 to *ABCC8*, but not to *KCNJ11* (Fig. 4B). Moreover, the mRNA levels of *USH1C* and *NCR3LG1*, two neighboring genes located in the same topologically associated domain (TAD) as this putative enhancer^35^, were not altered (Fig. 4C; *n* = 3 independent donors); likewise, *INS* mRNA levels did not significantly change (Fig. 4C; *n* = 4 independent donors). Consistent with these findings, patch clamping of single β-cells revealed a reduction in K_ATP_ currents following targeting of the enhancer region flanking rs1002226 in the *KCNJ11-ABCC8* locus (Fig. 4D; *n* = 3 independent donors). Thus, we achieved CRISPR-based targeting of non-coding regulatory regions in human β-cells, and our in vivo studies of a putative enhancer in native β-cells revealed a selective impact on the expression of *ABCC8* compared to *KCNJ11*.

**Fig. 5:**
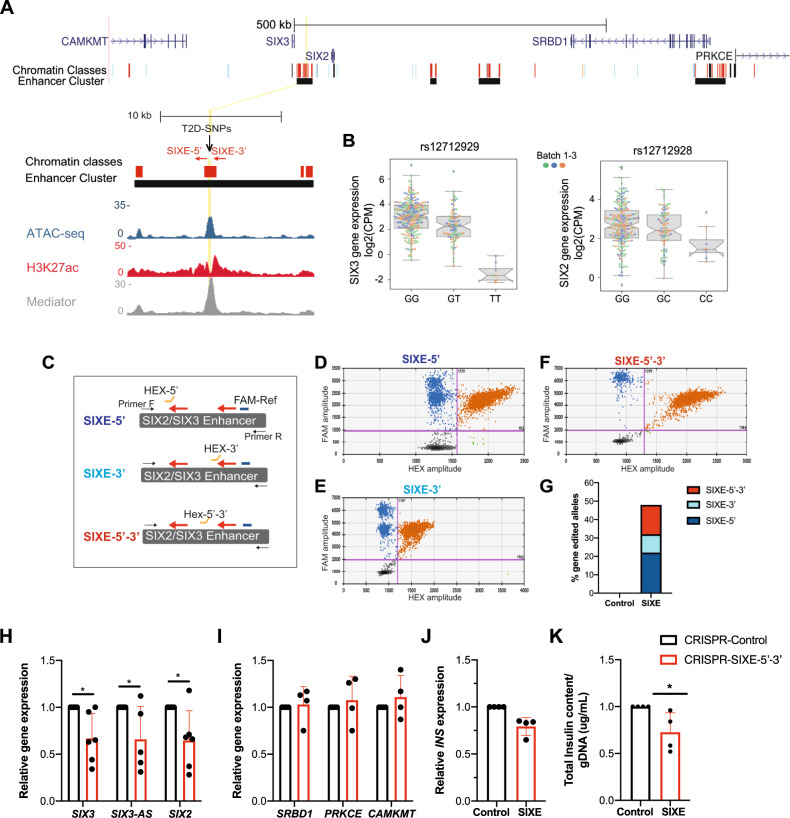
CRISPR/Cas9 targeting of a putative enhancer element in the *SIX2-SIX3* locus in primary human islets. **A** Genome Browser tracks of the genomic context in the *SIX2-SIX3* locus, highlighting the putative SIX2-SIX3 enhancer element (SIXE) with variants previously linked to increased risk of fasting glucose hyperglycemia and T2D (T2D-SNPs, black arrowheads): These variants map to an active enhancer within an enhancer cluster (yellow line, and zoomed inset); sgRNAs used for CRISPR-Cas9 targeting of the region flanking FG-SNP: SIXE-3′ and SIXE-5′ (red arrows). The two sgRNAs were cloned in the same construct (scheme in Fig. 4A). Chromatin classes: active promoter (green); active enhancer (red); inactive enhancer (gray); inactive open chromatin (black); strong CTCF (blue). Accessible chromatin regions in human islets are shown by ATAC-seq, H3K27ac and mediator ChiP-seq. **B** Islet eQTLs showing association of reduced expression levels of *SIX2* and *SIX3* and variants within the SIXE region targeted in this study (cis-eQTL mapping across 292 human islet samples, q-val= 4.9e−12 for rs12712929-TT and reduced level of *SIX3* and q-val= 0.008, for rs12712928 -CC and reduced expression of *SIX2*). Box plot shows the interquartile range (IQR) of 1st (Q25) and 3rd (Q75) quartiles, with the median as a black line in the center, and whiskers depict ± 1.5 times the IQR. For *SIX3* and GT, GT, and TT genotypes, Q25 values are 2.09, 1.41 and −2.03, and Q75 values are 3.92, 3.04, and −1.10, respectively. For *SIX2* and GG, GC and CC genotypes, Q25 values are 2.02, 1.89 and 1.27, and Q75 values are 3.42, 3.20, and 1.03, respectively. **C** Scheme of the ddPCR assay used to identify gene editing as consequence of targeting by SIXE-5′, SIXE-3′, or both (SIXE-5′−3′) in the pseudoislets targeted with SIXE-5′–3′. HEX probes are shown with an orange line, FAM reference probes, in blue **D**–**F** Example of ddPCR 2D plots showing GFP^+^ cells modified by (**D**) SIXE-5′, (**E**) SIXE-3′, (**F**) SIXE-5′–3′ (KO cells: FAM^+^/HEX^−^, blue droplets; wild-type droplets: FAM^+^/HEX^+^, orange, *n* = 2 independent donor samples). **G** Total percentage of gene-edited alleles in GFP^+^ cells, showing the contribution of (**D**–**F**) in the DNA of GFP^+^ islet cells. **H**–**J** mRNA levels of (**H**) *SIX3* (*n* = 6 independent donors; *P* = 0.0279), *SIX3-AS1* (*n* = 5 independent donors; *P* = 0.0437) and *SIX2* (*n* = 6 independent donors; *P* = 0.04), (**I**) control genes (*n* = 4 independent donor samples), (**J**) *INS* (*n* = 4 independent donor samples) in GFP^+^ cells targeted with CRISPR-SIXE-5′–3′. (**K**) Total insulin content of sorted GFP^+^ cells normalized to genomic DNA (gDNA) content (*P* = 0.0391; *n* = 4 independent donor samples). Data are presented as mean values ± SD. Two-tailed t tests were used to generate *P*-values. *P < 0.05. Source data are provided as a Source Data file.

LentiCRISPR-pseudoislet based methods could provide a powerful strategy for in vivo analysis of the regulation of genes that are active in human islets but not in other experimental models^10^. To assess this possibility, we targeted the *SIX2-SIX3* locus, which encodes the transcription factors *SIX2* and *SIX3*, and the *SIX3* antisense (*SIX3-AS1*) lncRNA^10^, that are expressed in human islet β-cells, but not in human cell lines, α-cells, or in mouse islets^10,11,40^. Nucleotide variants in the locus encoding *SIX2* and *SIX3* (Fig. 5A) have been linked by GWAS to increased risk for type 2 diabetes (T2D) and diabetes-related traits^41–44^. These variants cluster in a putative *SIX2-SIX3* enhancer element previously assessed with episomal luciferase assays in transgenic immortalized mouse β-cell lines^45^ (SIXE: Fig. 5A), but not in native human β-cells. We performed *cis*-expression quantitative trait loci (eQTL) mapping in 292 human islet samples (Methods), and found two variants within this putative enhancer, rs12712929 and rs12712928, showing strong association with islet eQTLs for *SIX2* and *SIX3*, in agreement with previously reported associations^45^. *SIX3* expression level was reduced in pancreatic islets with variant rs12712929-TT (q-val= 4.9e−12), while reduced islet expression of *SIX2* was strongly associated with rs12712928 -CC (q-val= 0.008: Fig. 5B, Supplementary Fig. 5A). However, our recent chromatin conformation studies of this locus^35^, including virtual 4C mapping (Supplementary Fig. 5B), did not detect high-confidence interactions between this SIXE element and *SIX2* or *SIX3* promoter regions. Thus, the in vivo genetic targets of the SIXE element remain to be established.

To evaluate this putative regulatory element in native β-cells, we transduced primary human islets with lentivirus (Methods) encoding *Cas9*, *GFP*, and two sgRNAs targeting enhancer sequences located 5′ (SIXE-5′) and 3′ (SIXE-3′) of the SNP cluster (Fig. 5C). After transduction and pseudoislet formation, we performed ddPCR on GFP^+^ cells (Methods; *n* = 2 independent donors) and quantified the specificity and extent of mutations induced with these sgRNAs. Transduction with SIXE-5′ led to indels at the site targeted by SIXE-5′ but not by SIXE-3′ (Supplementary Fig. 6A). Likewise, SIXE-3′ transduction produced indels only at the site targeted by SIXE-3′ and not SIXE-5′ (Supplementary Fig. 6B). Transduction with both SIXE-5′ and SIXE-3′ led to an average 48% mutation frequency, including indels at the site targeted by sgRNAS SIXE-5′ (22%), at the site targeted by SIXE-3′ (10%), or deletions between these sites (16%) (Fig. 5C–G). Measures of mRNA using qRT-PCR in purified GFP^+^ cells targeted with the lentiCRISPR-SIXE-5′–3′ (Fig. 5H), or SIXE-5′ sgRNA alone (Supplementary Fig. 6C) revealed significantly reduced levels of *SIX2*, *SIX3* or *SIX3-AS1* mRNA (*n* = 6 independent donors). Assessment of the correlation between the degree of SIXE-5′–3′ editing and the expression level of *SIX2*, *SIX3,* or *SIX3-AS1* suggests that the effect of SIXE targeting varied between human donor samples, as indicated by the range of gene expression changes noted (Supplementary Fig. 6E). Targeting of SIXE with the SIXE-3′ sgRNA or the non-targeting sgRNA did not significantly reduce levels of *SIX2*, *SIX3*, or *SIX3-AS1* (Fig. 5H, Supplementary Fig. 6C). Levels of other mRNAs from genes inside the same TAD, such as *CAMKMT* and *SRBD1*, or in adjacent TADs, such as *PRKCE*, were not affected by the SIXE-5′-3′ targeting (Fig. 5A, I, Supplementary Fig. 6D; *n* = 4 independent donors). We noted a modest reduction of average *INS* mRNA levels after SIXE-5′–3′ targeting (Fig. 5J), and total insulin content from GFP^+^ SIXE-5′–3′ cells was significantly lower compared to those from non-targeting sgRNAs (Fig. 5K). Thus, our CRISPR targeting data provide evidence that a non-coding genomic element functions as an enhancer in the *SIX2-SIX3* locus of human β-cells. Moreover, localized 5′ or 3′ targeting achieved here suggested asymmetrical structural features within the SIXE element regulating the expression of *SIX2* and *SIX3*. These findings indicate that in vivo enhancer structure in primary human islet cells can be probed using CRISPR-targeted genomic deletions.

### CRISPRa enhancer targeting induces expression of *SIX2* and *SIX3*

**Fig. 6:**
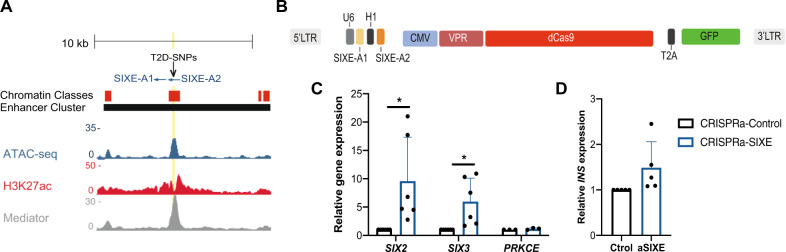
CRISPR/dCas9 activation of a putative enhancer element in the *SIX2-SIX3* locus in primary human islets. **A** Genome browser tracks highlighting the putative *SIX2*-*SIX3* enhancer element (SIXE) with variants previously linked to increased risk of fasting glucose hyperglycemia and T2D (T2D-SNPs, black arrowheads): These variants map to an active enhancer within an enhancer cluster (yellow line); sgRNAs used for CRISPRa: SIXE-A1, SIXE-A2. Chromatin classes: active enhancer (red); Accessible chromatin regions in human islets are shown by ATAC-seq, H3K27ac, and Mediator ChiP-seq. **B** Schematics of the lenti-construct used for simultaneous expression of two sgRNAs, VPR, dCas9 and GFP (13,728 bp) in primary human islets. **C**–**D** mRNA levels of (**C**) *SIX2* (*P* = 0.042; *n* = 6 independent donors), *SIX3* (*P* = 0.0328; *n* = 6 independent donors), *PRKCE* (*n* = 3 independent donors) and (**D**) *INS* (*n* = 5 independent donors), in GFP^+^ cells after CRISPRa of SIXE (CRISPRa-SIXE). Data are presented as mean values ± SD. Two-tailed t tests were used to generate *P*-values. *P < 0.05. Source data are provided as a Source Data file.

In addition to assessing the requirement for the SIXE element in regulating the expression of *SIX2* and *SIX3*, we evaluated the use of CRISPRa to activate this enhancer in primary human islet cells (Fig. 6A). We created a lentiviral construct that simultaneously expresses *dCas9-VP64-p65-Rta*^23^, a *GFP* transgene, and two sgRNAs for the SIXE enhancer (SIXE-A1 and SIXE-A2; CRISPRa-SIXE: Methods; Fig. 6B). After infection of primary dispersed human islet cells, pseudoislet development and subsequent purification of GFP^+^ cells (Methods), qRT-PCR revealed increased levels of both *SIX2* and *SIX3* mRNA after CRISPRa-SIXE compared to controls (Fig. 6C; *n* = 6 independent donors); the low abundance of *SIX3-AS1* mRNA precluded detection in these studies. Moreover, CRISPRa induction was specific for *SIX2* and *SIX3*: levels of mRNA encoding the neighboring gene *PRKCE* were indistinguishable in CRISPRa-SIXE and control samples (Fig. 6C). Thus, SIXE enhancer CRISPRa achieved simultaneous induction of *SIX2* and *SIX3* in native human β-cells. Consistent with prior studies suggesting that SIX2 regulates *INS* expression^10,22^, average *INS* mRNA levels were also modestly increased by CRISPRa-SIXE (Fig. 6D), though this change did not reach statistical significance. Together, our results with CRISPR targeting demonstrate that an enhancer in the *SIX2*-*SIX3* locus linked by GWAS to diabetes and diabetes-related traits is necessary and sufficient for in situ regulation of those genes in human β-cells. In sum, our studies with CRISPR/Cas9 and CRISPRa suggest that the architecture and function of non-coding genomic regions can be interrogated in native human islet cells.

## Discussion

CRISPR/Cas9-based genome editing remains a challenge in quiescent cells (reviewed in^46^) and has not been reported in adult pancreatic islet cells, which are post-mitotic^16,17^. Our study reveals index application of CRISPR/Cas9 and CRISPRa for gene editing and activation in primary human islet cells. Our results demonstrate that both coding exons and non-coding regulatory DNA can be mutated in primary human islet cells, resulting in altered gene expression and cellular function. We also demonstrate that targeting regulatory DNA with CRISPRa can induce the expression of genes in primary islet cells. CRISPR-based targeting has been reported in human stem cell-derived insulin-producing cells^47^ and in immortalized β-cell lines^35,48^ (EndoCβH3). Despite the value of these models, they are fundamentally different from genuine pancreatic islet cells in terms of gene regulation, maturation, function, proliferation, and cellular composition^49^. Thus, the relevance of conclusions from findings with these surrogate cell types to the biology and genetics of human islets remains limited^9,10^. Mutation or activation of regulatory DNA elements with CRISPR in *bona fide* human islets here achieved selective changes in expression of imputed target genes, and revealed unexpected structural features of islet enhancers. The application of these technologies in primary human islets provides new possibilities to understand genetic regulation of islet biology and disease.

Our findings provide a proof of concept that discrete coding and non-coding DNA elements can be targeted by CRISPR-based approaches in quiescent primary human pancreatic islet cells. In humans, mutations can lead to monogenic forms of diabetes. For example, loss-of-function heterozygous mutations in *PDX1* can lead to heritable early-onset diabetes^3^. We created targeted mutation of *PDX1* in primary human islet cells, and demonstrated for the first time how this could be used to model human islet β-cell phenotypes resulting from postnatal PDX1 loss. Prior studies in mice suggest that Pdx1 may promote β-cell survival in some contexts^50,51^, and PDX1 inactivation achieved here could allow future assessment of such roles in adult human β-cells. For the *KCNJ11* locus, we likewise demonstrated that our approach achieved loss-of-function phenotypes in postnatal human islets. Thus, approaches here should be useful for modeling diabetes genetics, including monogenic diabetes, which accounts for approximately 2% of human diabetes^52^.

CRISPR editing of non-coding DNA elements in human islet β-cells also provided evidence for the regulation of *ABCC8* and the *SIX2-SIX3* loci. Deletion within an enhancer element located in the *KCNJ11*-*ABCC8* locus led to reduced expression of one candidate target gene (*ABCC8*) but not the other (*KCNJ11*, which is more proximal to this enhancer), revealing an unsuspected in vivo function of this enhancer. In another example, CRISPR-targeted deletion in a single putative enhancer led to reduced expression of two nearby genes, *SIX2* and *SIX3*. Thus, CRISPR targeting in primary human islet cells revealed regulatory interactions not previously detected with chromatin conformation assays^24^. This result was corroborated and extended by the finding that sgRNA-based targeting of the 5′ region of the SIXE enhancer, but not the 3′ region, was sufficient to reduce *SIX2* and *SIX3* mRNA levels, providing evidence in human β-cells for structural asymmetry in this candidate enhancer. Prior studies^53^ have suggested *SIX2* and *SIX3* are positioned on opposite sides of a topologically associated domain (TAD), suggesting that regulatory effects of the SIXE enhancer might not extend to genes in an adjacent TAD. Our studies, however, suggest that the SIXE non-coding regulatory element regulates both *SIX2* and *SIX3*. Thus, our work demonstrates that interrogation with CRISPR is a powerful approach for identifying the function of regulatory elements in primary human islets, and is complementary to biochemical or imaging-based studies of chromatin conformation. With further improvements, CRISPR/Cas9 targeting also holds promise for in vivo targeted allele replacement in *bona fide* native human islet cells. If so, CRISPR/Cas9 targeting in primary islets would provide a consummate experimental means for deconvoluting mechanisms underlying the association of non-coding and coding variants to human type 2 diabetes genetic risk^37^. We speculate that the lentiCRISPR-pseudoislet strategy described here is also applicable to gene editing in human α cells and δ cells, whose genetic regulation and likely roles in diabetes remain relatively under-explored^54,55^.

## Methods

### Human Islet Procurement

Organs and islets were procured through the Integrated Islet Distribution Network (IIDP), National Diabetes Research Institute (NDRI) and the Alberta Diabetes Institute (ADI) Islet Core. De-identified human islets were obtained from healthy, non-diabetic organ donors with less than 18 h of cold ischemia time, and deceased due to acute trauma or anoxia. For this study, islets from 30 adult donors were used (Supplementary Table 1).

### Constructs and lentivirus production

pRSGCCG-U6-sg-CMV-Cas9-2A-TagGFP2 lentiviral constructs coding for Cas9, TagGFP2, and a sgRNA targeting hPDX1, hKCNJ11, or with a non-targeting sequence as control, were obtained from Cellecta Inc. For dual expression of two sgRNAs, pRSGCCG-U6-empty-CMV-Cas9-2A-TagGFP2 was digested with *Bbs*I and *Xho*I and the scaffold for a second sgRNA was HiFi fused, resulting in pRSGCCG-U6-scaffold-CMV-Cas9-2A-TagGFP2-dual. The cloning of two sgRNAs and the H1 promoter was performed as previously described^39^. For the generation of an all-in-one-CRISPRa-dual plasmid, VPR-dCas9 was amplified from pHR SFFV-VPR-dCas9-P2A-Cherry (kindly provided by the Stanley Qi Lab^14^) and HIFI fused to amplified pRSGCCG-U6-empty-CMV---2A-TagGFP2-dual, resulting in pRSGCCG-U6-empty-CMV-VPR-dCas9-2A-TagGFP2-dual. The selection of the enhancer in the KCNJ11-ABCC8 locus was based on findings from pcHi-C^35^ showing putative associations of this T2D risk variant. The putative enhancer in the *SIX2-SIX3* locus was initially described by^45^. The sgRNAs used in this study were designed using E-CRISPR and MIT CRISPR design tool. All sgRNA sequences used in this study can be found in Supplementary Table 2.

Lentiviruses were produced by transient transfection of HEK293T cells with lentiviral constructs, and with pMD2.G (12259; Addgene) and psPAX2 (12260; Addgene) packaging constructs. Mirus TransIT reagents were used for transfection (Mirus Bio LLC), according to manufacturer recommendations. Supernatants were collected and purified using PEG-it (System Biosciences). Concentrated lentivirus was stored at −80 °C until transduction of primary human cells.

### Human pseudoislet generation and lentiCRISPR transduction

Human islets were dispersed into a single cell suspension by enzymatic digestion (Accumax, Invitrogen). For each experimental condition, ~1 × 10^6^ cells were transduced with lentivirus corresponding to 1 × 10^9^ viral units in 1 ml as determined by the Lenti-X qRT-PCR titration kit (Clonetech), in medium supplemented with 8 ug/ml Polybrene. At the time of dispersion and transduction, we exposed cells to the small molecule GNF4877, a DYRK1A inhibitor (MCE: MedChemExpress). Mean transduction efficiencies, determined by GFP^+^ cell quantification, were slightly higher for cells exposed to GNF4877 (2 uM) at the time of infection (41%) compared to controls (34%) (Supplementary Fig. 7A). With TIDE PCR, indels were detected in an average of 57.4% of PDX1 sequences in the absence of GNF4877 (Supplementary Fig. 7B; *n* = 3 independent donors). Using ddPCR, 30.2% mutated alleles were detected for CRISPR-PDX1 without initial exposure to GNF4877 (Supplementary Fig. 7C; *n* = 2 independent donors). While prolonged exposure to GNF4877 can induce β-cell proliferation^56^, its effect alone is not significant^57^. Here, after transient GNF4877 exposure, we did not detect increased production of the proliferation marker MKI67 (Supplementary Fig. 7D). Moreover, reduction of *PDX1* mRNA measured by RT-qPCR revealed no difference in outcomes with or without GNF4877 exposure (Supplementary Fig. 7E).

For CRISPRa experiments, TransDux MAX Lentivirus Transduction Reagent was used for infection, per the manufacturer’s specifications (System Biosciences). Transduced islet cells were cultured in ultra-low attachment 96-well plates (Corning) for 4 days at 37 °C with 5% CO_2_ in culture medium consisting of RPMI 1640 (Gibco), 2.25 g/dl glucose, 1% penicillin/streptomycin (v/v, Gibco), and 10% fetal bovine serum (HyClone). On day 4, pseudoislets in all CRISPR/Cas9 experiments were transferred to a 6 well plate in culture medium comprised of RPMI 1640 (Gibco), 2.25 g/dl glucose, 1% penicillin/streptomycin (v/v, Gibco), and 2% human serum (Sigma-Aldrich), and cultured until day 6 prior to further molecular or physiological analysis. For CRISPRa experiments, pseudoislets were evaluated on day 4.

### FACS sorting

Pseudoislets were dispersed into single cells by brief enzymatic digestion (Accumax, Invitrogen). Cells were stained with LIVE/DEAD™ Fixable Near-IR Dead Cell Stain Kit (Life Technologies). Non-GFP^+^ cells were used as controls. Cells were sorted on a special order 5-laser FACS Aria II (BD Biosciences) using a 100 µm nozzle, with doublet removal. Sorted cells were collected into low retention tubes containing 50 µL of FACS buffer (2% v/v fetal bovine serum in phosphate-buffered saline) supplemented with Ribolock RNase inhibitor (Thermo Scientific). DNA, RNA, and/or protein were isolated from sorted GFP^+^ cells. Cytometry data was analyzed and graphed using FlowJo software (TreeStar v.10.8).

### Genomic DNA extraction, TIDE, and ddPCR analysis

1000–5000 GFP^+^ cells were used for genomic DNA extraction using the Arcturus^®^ PicoPure^®^ DNA Extraction Kit (Thermo Fisher Scientific) per the manufacturer’s instructions. 4 ul of extracted genomic DNA were used as PCR template. PCR was performed with Accuprime Pfx (Thermofisher) in the presence of 4 M betaine (Sigma-Aldrich) in 40 cycles, each cycle composed of a denaturing step at 95 °C for 30 s, an annealing step at 60 °C for 40 s for PDX1, 62 °C for 1 min for KCNJ11, and an extension step at 68 °C for 30 s, followed by a final extension at 68 °C for 5 min. PCR amplicons were purified with DNA Clean and Concentrator Kit (Zymo Research, Irvine, CA) for sequencing and subsequent TIDE analyses to assess indel efficiency. For ddPCR, a whole-genome amplification step using Repli-g midi kit (Qiagen), was performed. QX200 ddPCR machine (Bio-Rad) was used following the manufacturer’s specifications. Data were analyzed using QuantaSoft Software (Bio-Rad). Primer and probes sequences are summarized in Supplementary Table 3.

### Evaluation of CRISPR/Cas9 off-target effects; RNA extraction, quantitative RT-PCR

Off-target site prediction was performed using the CHOP-CHOP tool^33^. The potential off-target sites found had at least 2 mismatches with respect to the sgRNA sequence. PCR primers encompassing 4 of these potential off-target sites were designed and the PCR amplicons were purified and sequenced. PCR was performed with Accuprime Pfx (Thermofisher) in the presence of 4 M betaine (Sigma-Aldrich) in 35 cycles, each cycle composed of a denaturing step at 95 °C for 1 min, an annealing step at 60 °C for 1 min and an extension step at 68 °C for 1 min, followed by a final extension at 68 °C for 5 min. Primer sequences are summarized in Supplementary Table 3.

RNA was isolated from GFP^+^ pseudoislet cells using the PicoPure RNA Isolation Kit (Life Technologies). cDNA was synthesized using the Maxima First Strand cDNA synthesis kit (Thermo Scientific) and gene expression was assessed by PCR using the Taqman Gene Expression Mix (Thermo Scientific). Low recovery of mRNA from some pseudoislet preparations after PDX1-CRISPR precluded assessment of some gene expression. Data were analyzed using Prism 6.0 h (GraphPad Software Inc., San Diego, CA). Paired two-tailed t tests were used to indicate statistical significance, and data are presented as mean and standard deviation.

### Perifusion, insulin, and glucagon content measurement, and Immunohistochemistry

Human islet perifusion experiments were performed using a 4 chamber Biorep Perifusion System following the manufacturer’s instructions. Equal batches of 100 human pseudoislets from CRISPR-Control and CRISPR-PDX1 from three independent donors were perifused at a flow rate of 100 ul/min for a total of 142 min in perifusion buffer with 5.6 mM, 16.7 mM and 16.7 mM +IBMX glucose concentrations. High glucose (16.7 mM) stimuli were applied for 30 min and glucose (16.7 mM) + IBMX was applied for 12 min. Fractions of perifusate were collected every 3 min. The first 10 fractions of each experiment were used to equilibrate islets in the perifusion system and discarded (30 min wash). For each experiment, a total of 30 fractions per condition were collected.

To determine total cellular insulin or glucagon content, both intact transduced pseudoislets (*n* = 15 pseudoislets, 4 independent donors) and sorted GFP^+^ cells (4000 cells, 3 independent donors) from CRISPR-PDX1 versus CRISPR-Control pseudoislets and sorted GFP^+^ cells (4000 cells, 3 independent donors) from CRISPR-SIXE versus CRISPR-Control pseudoislets were sonicated and lysed to extract the total cellular insulin or glucagon content (Human insulin and glucagon ELISA kits, Mercodia). Data were analyzed using Prism 6.0 h (GraphPad Software Inc., San Diego, CA), normalized to the CRISPR-Control and presented as mean with standard deviation. Paired two-tailed t tests were used to indicate statistical significance.

For immunostaining, human pseudoislets were fixed for 1 h at 4 °C and embedded in collagen (Wako Chemicals); mouse pancreata were fixed in 4% paraformaldehyde overnight at 4 °C. Ten micrometers thick frozen sections were cut and stained following standard cryostaining protocols. Briefly, sections were washed in PBS, incubated with blocking solution followed by incubation in permeabilization/blocking buffer (1% bovine serum albumin, 0.2% non-fat milk, 0.5% Triton-X in PBS) for 1 h. Primary antibodies were mixed with permeabilization/blocking buffer and incubated at 4 °C overnight. The following primary antibodies were used: goat anti-PDX1, (1:200; Sigma-Aldrich 06-1385), Guinea pig anti-insulin (1:500, a0564 Dako), mouse anti-glucagon (1:500, G2654 Sigma), Alexa Fluor 555 Goat anti Guinea Pig IgG (H + L) Secondary Antibody (A21435; Invitrogen) (1:500, Invitrogen), Alexa Fluor^®^ 647-AffiniPure Donkey Anti-Mouse IgG (H + L) (715-605-150; Jackson ImmunoResearch) (1:500). Slides were washed with PBS, incubated with secondary antibodies at room temperature for 2 h, and were preserved with mounting medium containing DAPI (Vector Labs, Vectashield H-1200). Images were obtained using a Leica SP2 confocal microscope.

### Transplantation and in vivo assessment of pseudoislet function

Human pseudoislets were transduced and cultured as described above. Batches of 300 pseudoislets were resuspended in cold Matrigel and transferred into the left renal capsular space of host animals using a glass micro-capillary tube. Transplant recipients were 8-week-old male NOD scid IL2Rγnull mice (stock number 005557; The Jackson Laboratory) and were anesthetized using ketamine/xylazine. Appropriate depth of anesthesia was confirmed by lack of toe-pinch response. One-month post-transplantation, mice were administered an intraperitoneal glucose injection at a dosage of 3 g/kg body weight. Glucose measurements and blood samples were collected via the tail vein at 0, 15, 30, 45, 60, 120, and 180 min post glucose injection. Human insulin is distinguishable from mice insulin, which allowed us to measure its levels by a human insulin ELISA kit (Mercodia).

### Patch-clamp studies

Single-cell patch-clamp studies were performed as described previously^58^. Briefly, human pancreatic pseudoislets were dissociated to single cells and cultured in low glucose conditions (5.5 mmol/L) for 1-3 days. Before starting whole-cell patch clamping, media was changed to bath solution containing (in mM): 118 NaCl, 20 Tetraethylammonium-Cl, 5.6 KCl, 1.2 MgCl2, 2.6 CaCl2, 5 HEPES, and 5 glucose (pH 7.4 with NaOH) in a heated chamber (32–35 °C). For patch-clamping, fire polished thin wall borosilicate pipettes, which were coated with Sylgard (3-5 MOhm), contained intracellular solution with (in mM): 125 Cs-glutamate, 10 CsCl, 10 NaCl, 1 MgCl2, 0.05 EGTA, 5 HEPES, 0.1 cAMP, and 3 MgATP (pH 7.15 with CsOH). Electrophysiological measures were collected using a HEKA EPC10 amplifier and PatchMaster Software (HEKA Instruments Inc, Lambrecht/Pfalz, Germany) within 5 min of break-in. Quality control was assessed by the stability of seal (>10 GOhm) and access resistance (<15 MOhm) over the course of the experiment. Data were analyzed using FitMaster (HEKA Instruments Inc) and Prism 6.0 h (GraphPad Software Inc., San Diego, CA).

### Expression quantitative trait loci (eQTLs)

To identify genetic effects on islet gene expression, we compiled 191 publicly available genotype and RNA-seq samples^59,60^ (GEO and EGA accession numbers GSE50244 and EGAS00001001265) and 101 in-house samples from human islet donors without diagnosis of diabetes (after QC analysis). cis-eQTL mapping across 292 human islet samples was performed using QTLtools^61^ and a cis-window of 500 kb up- and downstream of the transcription start site (TSS). 10 principal components (PCs) derived from gene expression and 4 genetic PCs were used as covariates in the linear model. Genetic PCs and population structure characterization were calculated using flashPCA^62^ on a subset of genotyped SNPs that were common in all 4 cohorts, with MAF ≥ 1% and missingness < 5% across all 292 samples. We also excluded SNPs in high LD (pairwise r2 ≤ 0.1 within 1 Mb window), C/G and A/T SNPs to avoid strand mismatches, and SNPs located in previously reported regions with long-range LD. We aggregated 1000 Genomes Phase3 reference dataset using overlapping variants to further characterize population structure. Gene expression PCs were calculated using prcomp on normalized counts. To identify the best associated cis eQTL SNP-eGene pairs, QTLtools was run using the permutation pass mode (1000 permutations), and beta approximated permutation *P*-values were adjusted for multiple testing correction using Storey q-values implemented in the qvalue R package^63^. The significance threshold was set at q-value ≤ 0.01.

### Study approval

Animal experiments were approved and performed in accordance with the guidelines provided by the Stanford University Institutional Animal Care and Use Committee (IACUC).

### Data visualization

Browser tracks were made with the UCSC genome browser. The graphics were made with BioRender.

### Reporting summary

Further information on research design is available in the Nature Research Reporting Summary linked to this article.

## Supplementary information

Supplementary Information

Reporting Summary

## Data Availability

The data that support the findings of this study are available from the authors on reasonable request, see author contributions for specific data sets. Source data are provided with this paper.

## Source data

Source Data
