# Supplementary material for: CRISPR-based genome editing in primary human pancreatic islet cells
Source: Nat Commun. 2021 Apr 23;12:2397. doi: 10.1038/s41467-021-22651-w (PMC8065166; doi:10.1038/s41467-021-22651-w)
Supplement: Supplementary file 1 — Supplementary Information [file 41467_2021_22651_MOESM1_ESM.pdf]

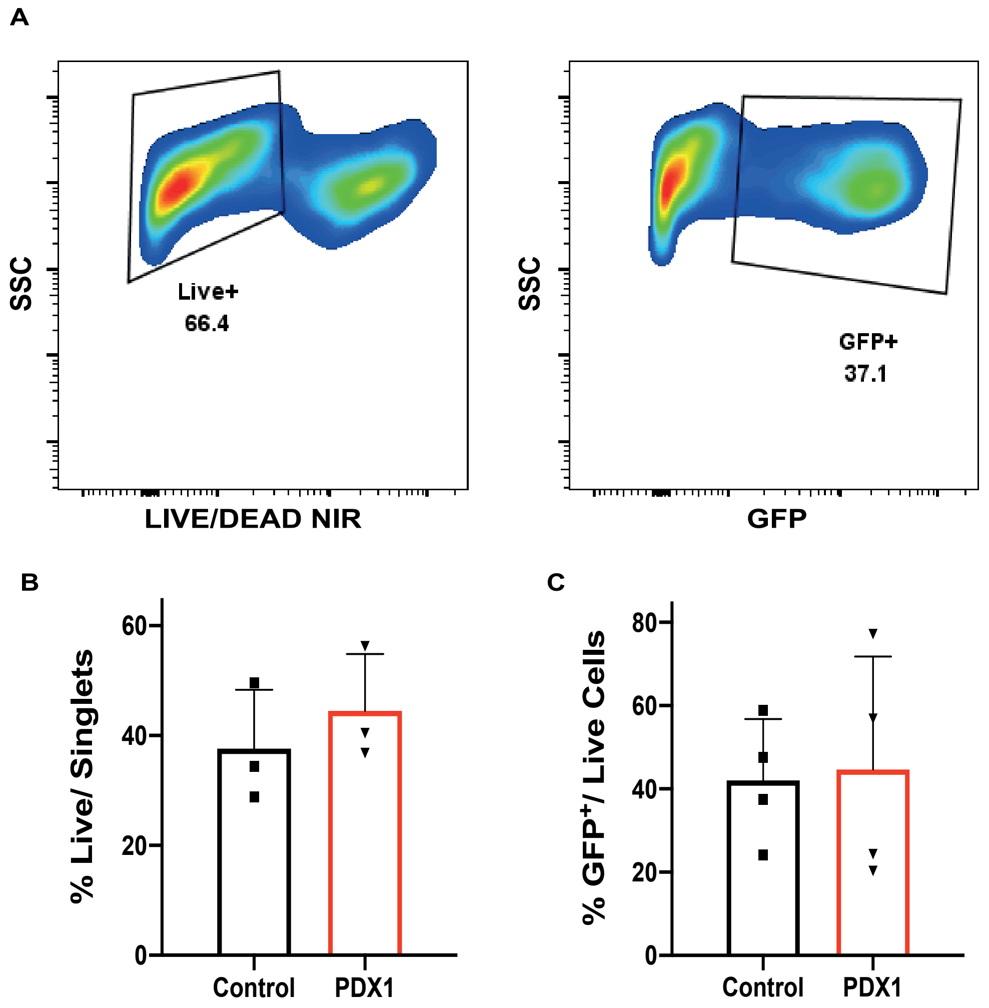

Supplemental Figure 1. Transduction efficiency of lentiCRISPR/Cas9 in primary human islets. **(A)** Example of FACS plots showing gates used to sort LIVE^{+}/GFP^{+} pseudoislet cells 6 days post-infection. **(B)** Proportion of live cells six days after lentiCRISPR infection of human pseudoislets (n= 3 independent donors). **(C)** Proportion of GFP^{+}/live cells for CRISPR-Control versus CRISPR-PDX1 infected pseudoislets (n= 4 independent donors). Data are presented as mean values +/- SD. Source data are provided as a Source Data file.

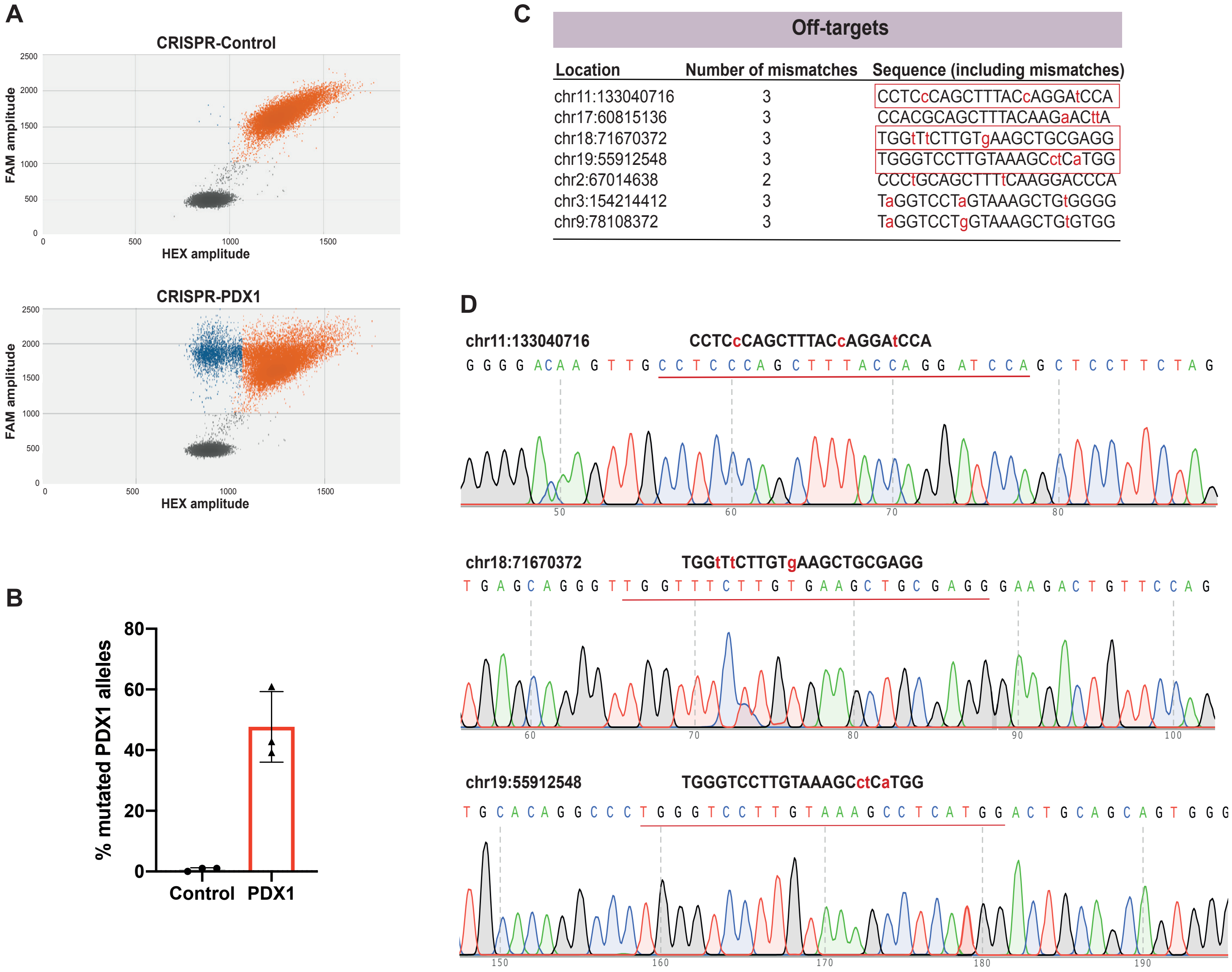

Supplemental Figure 2. CRISPR/Cas9 targeting of PDX1 protein-coding sequence in primary human islets (A-B) ddPCR was used to assess the efficiency of PDX1 targeting in DNA extracted from CRISPR-Control and CRISPR-PDX1 GFP+ human islet cells 6 days after infection: wild-type droplets (orange, HEX+/FAM+); KO doplets (blue: HEX-/FAM+). **(A)** Example of ddPCR 2D plot for CRISPR-Control and CRISPR-PDX1 GFP+ human islet cells. **(B)** Proportion of PDX1 mutated alleles for CRISPR-Control and CRISPR-PDX1 (n=3 independent donors). Data are presented as mean values +/- SD. Source data are provided as a Source Data file. **(C)** Bioinformatics assessment of potential off-target genomic sites using CRISPR/Cas9 and our sgRNA targeting PDX1 exon 1: PCR-amplification followed by sequencing of genomic DNA extracted from CRISPR-KCNJ11 GFP+ cells confirmed these potential off-target sites showed wild-type sequences. Red boxed, sequences shown as example in (D). **(D)** The sequence of three of these sites are exemplified here, showing wild-type sequences at the predicted off-target sites.

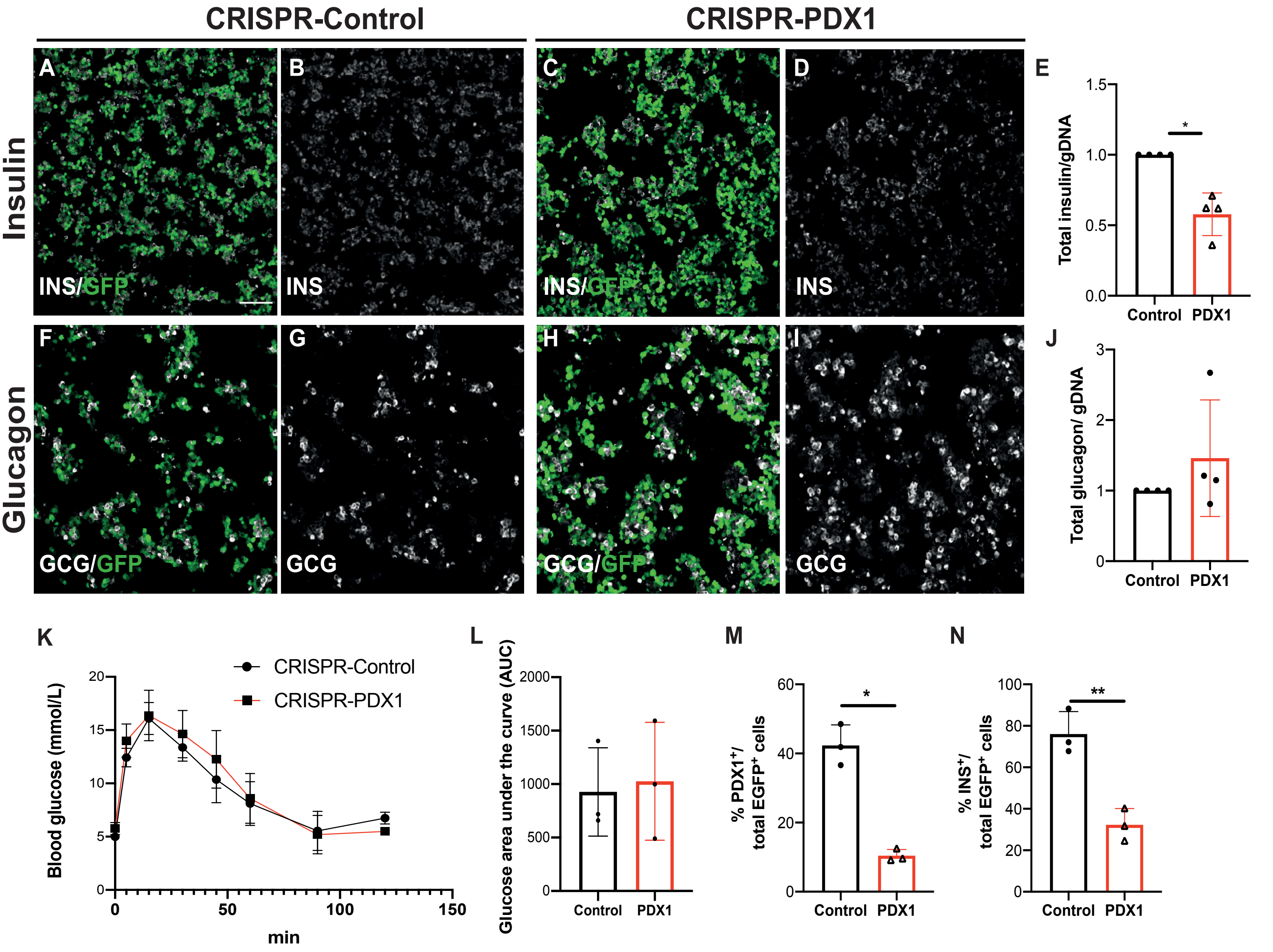

Supplemental Figure 3. Insulin and glucagon content in CRISPR-PDX1 pseudoislets. (A-D) Insulin immunostaining of human pseudoislets following infection with **(A-B)** CRISPR-Control versus **(C-D)** CRISPR-PDX1. GFP, green; INS, grey (n=2 independent donors). **(E)** Total insulin content from lysates of CRISPR-Control (Ctrol) versus CRISPR-PDX1 (PDX1) intact human pseudoislets six days after lenti-CRISPR infection (*P*=0.0114; n= 4 independent donors). **(F-I)** Glucagon immunostaining of human pseudoislets following infection with **(F-G)** CRISPR-Control versus **(H-I)** CRISPR-PDX1. GFP, green; GCG, grey (n= 2 independent donors). **(J)** Total glucagon content from lysates of CRISPR-Control versus CRISPR-PDX1 (PDX1) intact human pseudoislets six days after lentiCRISPR infection (n= 4 independent donors). **(K)** Blood glucose levels of mice transplanted with CRISPR-Control versus CRISPR-PDX1 pseudoislets, measured at the time of IPGTT (n=4 mice/condition; 3 independent human islet donors). **(L)** Area under the curve of blood glucose levels shown in (**K**). (**M**) Quantification of GFP$^+$ PDX1$^+$ cells from immunostaining sections from grafts recovered from mice transplanted with CRISPR-Control versus CRISPR-PDX1 pseudoislets (P=0.0137; n=3 independent donors- recovered grafts). (**N**) Quantification of GFP$^+$ INS$^+$ cells from immunostaining sections from grafts recovered from mice transplanted with CRISPR-Control versus CRISPR-PDX1 pseudoislets (P=0.0026; n=3 independent donors- recovered grafts). Data are presented as mean values +/- SD for Supplemental 3E,J,L-N and as mean values +/- SEM for Supplemental 3K. Two tailed t-tests were used to generate *P* values. *$P<0.05$. Scale bar: 50 um. Source data are provided as a Source Data file.

A

Off-targets		
**Location**	**Number of mismatches**	**Sequence (including mismatches)**
chr10:83187436	3	CCAtTGATCATCTAaCATaTCAT
chr14:94968523	3	ATcACATGGTAGAgGAgCAGAGG
chr22:23095906	3	ATGgCATtGTAGATGATgAGGGG
chr3:63676059	3	ATGACcTtGTAGATGATtAGTGG
chr5:67354417	3	CCTCTGATCAcCTACCAgcTCAT
chr5:111071627	3	ATGACATGGTAGAaaATCAtTGG

B

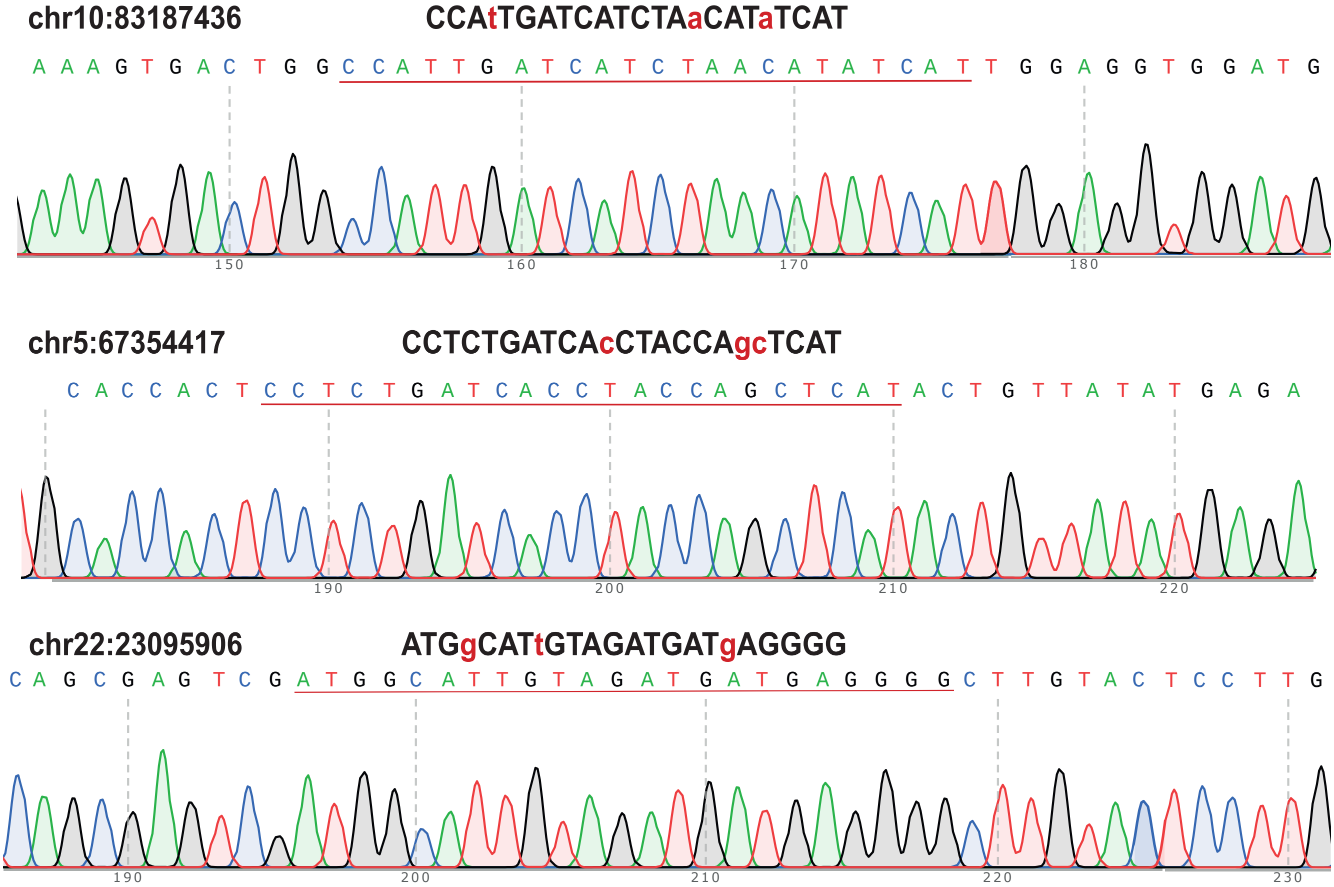

Supplemental Figure 4. Assessment of potential off-target sites using CRISPR/Cas9 and a sgRNA targeting KCNJ11 exon 1 in primary human islets (A) Potential off-target genomic sites identified using bioinformatics for CRISPR/Cas9 and our sgRNA targeting KCNJ11 exon 1: PCR-amplification followed by sequencing of genomic DNA extracted from CRISPR-KCNJ11 GFP^{+} cells confirmed these potential off-target sites showed wild-type sequences. Red boxed, sequences shown as example in (B). **(B)** The sequence of three of these sites are exemplified here, showing wild-type sequences at the predicted off-target sites.

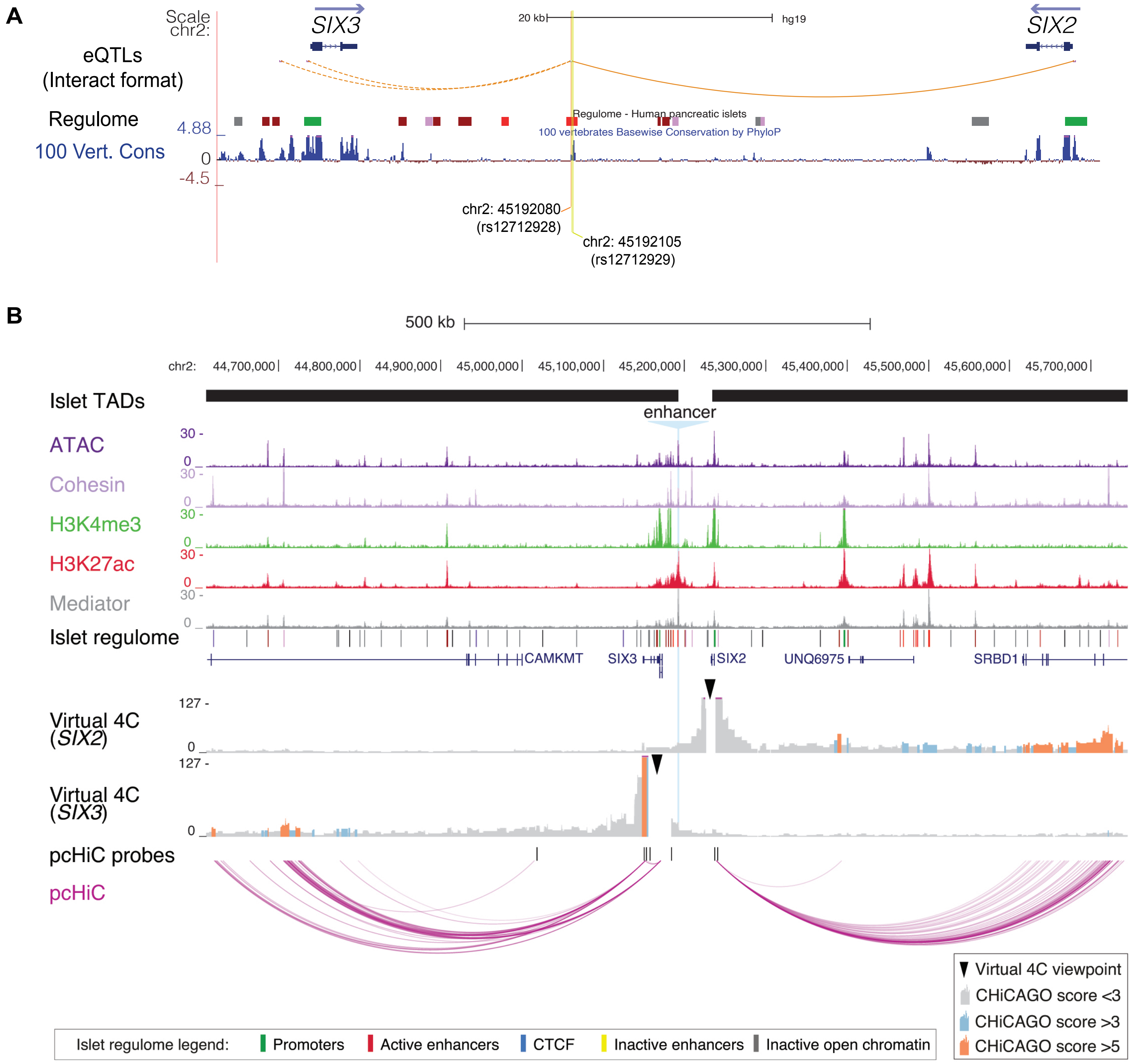

Supplemental Figure 5. Variants within the putative enhancer element SIXE are associated with cis eQTL for *SIX2* and *SIX3*, while pcHiC is unable to detect chromatin interactions between this SIXE element and SIX2 or SIX3 promoter regions in primary human islets. (A) cis-eQTL mapping across 292 humam islet samples identified best associated cis eQTL SNP-eGene pairs (q-value ≤ 0.01). **(B)** Epigenomic maps and 3D chromatin interactions in human islets in the SIX2/SIX3 locus. Virtual 4C representations show low-confidence interactions (grey) between SIXE element (highlighted in blue) and SIX2 and SIX3 promoters. High-confidence interactions (CHICAGO score>5) are represented in orange in virtual 4C and as pink arcs (bottom track). The HindIII fragments that contain SIX2 or SIX3 genes are used as viewpoints and depicted as inverted triangles.

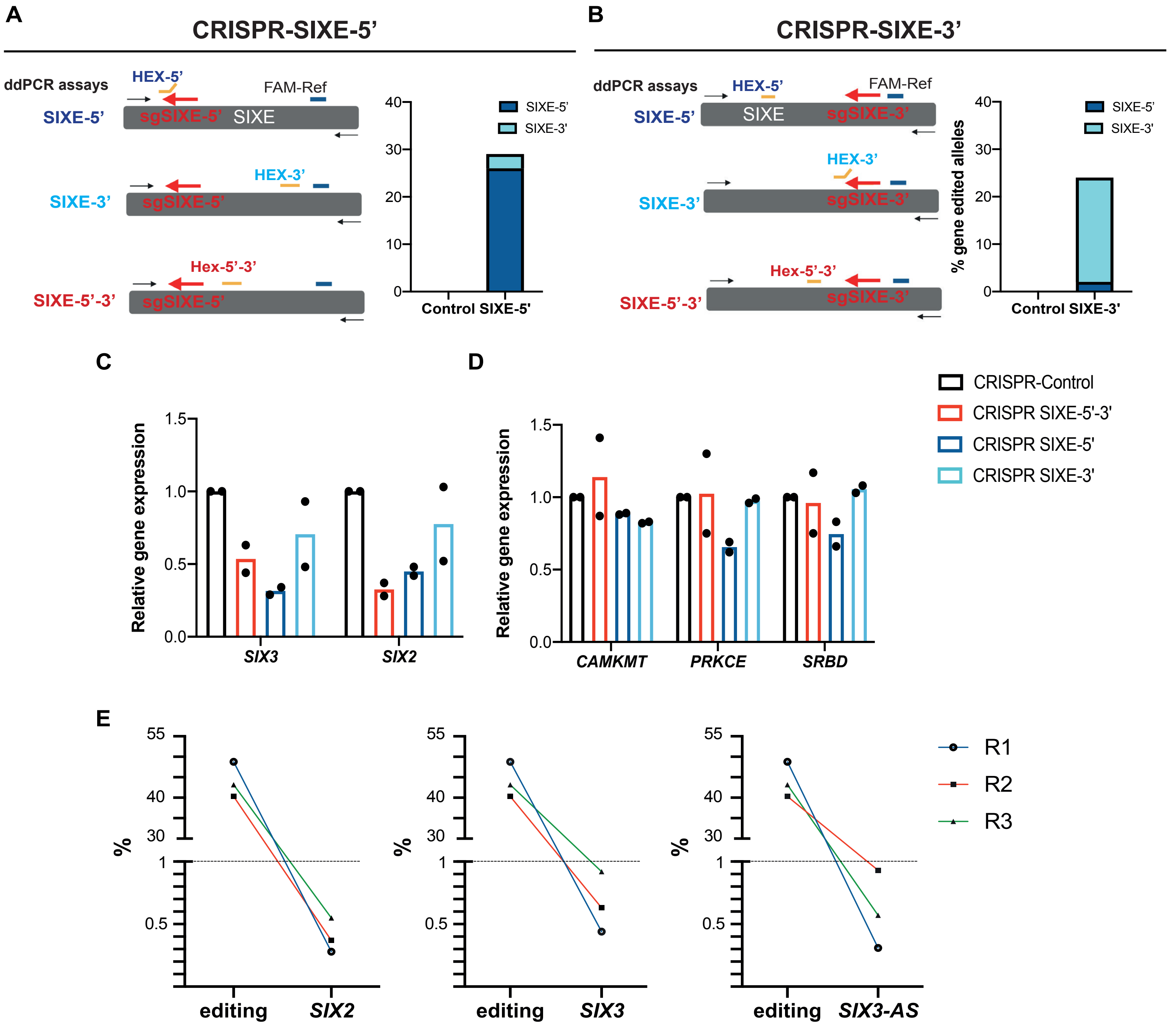

Supplemental Figure 6. Relates to Figure 5: CRISPR/Cas9 targeting of a putative enhancer element in the *SIX3-SIX2* locus in primary human islets. (A-B) Scheme of ddPCR assays used to detect the total percentage of gene-edited alleles in GFP⁺ cells transduced with **(A)** lentiCRISPR constructs coding for the sgRNA SIXE-5’ or **(B)** SIXE-3’. In both cases, the total % of gene edited alleles is shown (n= 2 independent donors). **(C-D)** mRNA levels of **(C)** *SIX2*, *SIX3* and **(D)** neighboring genes, in GFP⁺ cells targeted with CRISPR-SIXE-5’-3’, or with CRISPR-SIXE-5’ or CRISPR-SIXE-3’ only. SIXE: SIX2-SIX3 putative enhancer (n= 2 independent donors). **(E)** Correlation between gene editing and gene expression of *SIX2*, *SIX3* and *SIX3-AS* following CRISPR-SIXE-5’-3’ across three repetitions (R1-R3) (n= 3 independent donors). Source data are provided as a Source Data file.

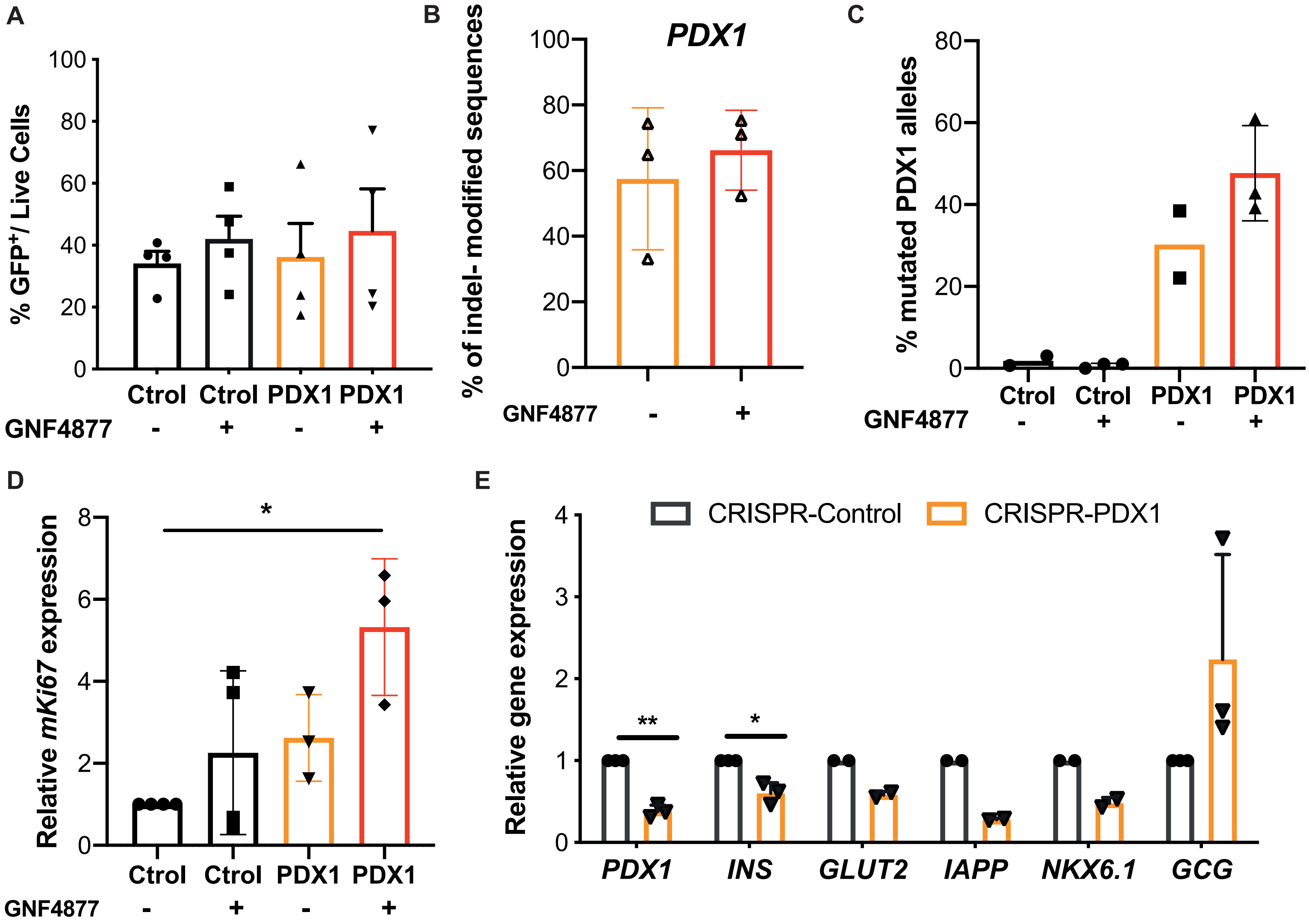

Supplemental Figure 7. Related to Methods: CRISPR/Cas9 targeting of PDX1 in primary human islets, assessment of supplementation with GNF4877 during infection. (A) Mean transduction efficiencies, determined by GFP⁺ cell quantification, of CRISPR-Control and CRISPR-PDX1, 6 days post-exposure to GNF4877 at the time of infection (n= 4 independent donors). **(B)** Detection of indels in DNA extracted from GFP⁺ cells using TIDE PCR, after exposure to GNF4877 at the time of infection (n= 3 independent donors). **(C)** Detection of indels in DNA extracted from GFP⁺ cells using ddPCR, comparison between addition or not of GNF4877 at the time of infection (n=2 or n=3 independent donors respectively). **(D)** Measurement of *MKI67* levels after exposure to GNF4877 at the time of infection (P=0.0462; n= 3 independent donors). **(E)** mRNA levels of PDX1-predicted targets, showing no difference in outcomes with or without GNF4877 exposure (refer to Fig. 2B; n= 3 independent donors for *PDX1* (P=0.0049), *INS* (P=0.034), *NKX6.1* and *GCG* and n=2 independent donors for *GLUT2* and *IAPP*). Data are presented as mean values +/-SD. Two tailed t-tests were used to generate P values. *P<0.05, **P<0.01. Ctrol: Control. Source data are provided as a Source Data file.

Supplemental Table 1. Details of donors used in this study.

Islet source	Identification	Sex	Age	BMI
IIDP	SAMN11963659	Male	52	31.6
IIDP	SAMN12227196	Male	51	32.8
ADI	SAMN12496597	Female	39	24.2
ADI	SAMN12731217	Male	30	25.5
ADI	SAMN12828004	Male	42	30
ADI	SAMN13440885	Female	64	20.4
IIDP	SAMN12274306	Male	37	25.3
IIDP	SAMN12500521	Male	52	29
IIDP	SAMN12633894	Female	57	32.9
ADI	SAMN12574735	Male	64	29.6
IIDP	SAMN12670838	Male	40	30.7
IIDP	SAMN13028024	Female	57	23.5
	AHA4312	Male	33	33
IIDP	SAMN14120450	Male	37	31.9
IIDP	SAMN14132340	Male	31	27
ADI	SAMN14291396	Male	54	29.4
IIDP	SAMN13866285	Female	30	27.1
UCSF	rHiP-141	Female	48	28.1
ADI	SAMN13850698	Male	79	23.5
ADI	SAMN13141063	Female	43	16.4
ADI	SAMN14052572	Male	64	24.3
IIDP	SAMN15314807	Male	27	25.3
ADI	SAMN16885745	Male	67	24.5
ADI	SAMN16956007	Male	48	25.7
IIDP	SAMN16191825	Male	35	31.3
ADI	SAMN17762820	Female	28	31.4
SDRC	AIA3480	Female	44	23.8
SDRC	AHJO226	Male	37	25
UCSF	rHIP-145	Female	55	31.8
IIDP	SAMN17528599	Male	60	29.9

Supplemental Table 2. Sequence of sgRNAs used in this study

Identification	Sequence
sgPDX1	TGGGTCCTTGTAAAGCTGCG
sgKCNJ11	ATGACATGGTAGATGATCAG
sg_EK1	TACTTCGGCAGAGAAAAATC
sg_EK2	GCCACAGAGAAGACTTACTT
sg_SE-5’	GCTGAGCCAGGCAGGGAGGC
sg_SE-3’	GGAGGAGTGCGAGCACAGCC
sg_SE-A1	CAGTGCTCCAGCCTCCCTGC
sg_SE-A2	GCACTCCTCCTGCCGATTAG

Supplemental Table 3. Sequence of primers and probes used in this study

Identification	Sequence
PDX1-E1-1F	GCCACACAGTGCCAAAT
PDX1-E1-1R	GAGGTGGTGGTGAAGGT
ddPDX1-1F	GCCACACAGTGCCAAATC
ddPDX1-1R	CCATGTACAGGCACGCA
PDX1-HEX probe	ACGCAGCTTTACAAGGACCCAT
PDX1-FAM probe	CTCCAGCTCCCGACTCCCGG
KCNJ11-CDS-1F	TGCTTCATGCTACGTGTG
KCNJ11-CDS-2F	ATCATCAGCGCCACCATC
KCNJ11-CDS-1R	CGTCCTCCTCAGCTACAA
KCNJ11-CDS-2R	TTTCCACCACGCCTTCC
KE-1F	CTCTCTATGCCTGGAGCTTTC
KE-1R	AGCCCAAACCAGAGTAACC
ddSE-1F	GGGTCTGTGTTCCGTGTG
ddSE-1R	TAATGCTCAACACTGGCTCA
ddSE-5’-HEX probe	CCAGTGCTCCAGCCTCCCTG
ddSE-3’-HEX probe	CCGGGCTGTGCTCGCACTCC
ddSE-5’-3’D-HEX probe	CCCTGCATTGTTTGCTGTCCTGAA
ddSE-FAM probe	TTTCAGGAGCCGAATGAGCTGAGC
dd_norm-HEX probe	CCCCATCTGTATGGTGGC
dd_norm-FAM probe	AGGTGTGAAGGTGCAAGCTCAGA
dd_norm-1F	GATCCCTAAGAGGACGAAGA
dd_norm-1R	CACCCTCACTGTATGTTGTC
