# Supplementary material for: CRISPR-based genome editing in primary human pancreatic islet cells
Source: Nat Commun. 2021 Apr 23;12:2397. doi: 10.1038/s41467-021-22651-w (PMC8065166; doi:10.1038/s41467-021-22651-w)
Supplement: Supplementary file 2 — Reporting Summary [file 41467_2021_22651_MOESM2_ESM.pdf]

nature research

Corresponding author(s): Seung K. Kim

Last updated by author(s): Mar 20, 2021

Reporting Summary

Nature Research wishes to improve the reproducibility of the work that we publish. This form provides structure for consistency and transparency in reporting. For further information on Nature Research policies, see our Editorial Policies and the Editorial Policy Checklist.

Statistics

For all statistical analyses, confirm that the following items are present in the figure legend, table legend, main text, or Methods section.

n/a	Confirmed	
☐	☒	The exact sample size (n) for each experimental group/condition, given as a discrete number and unit of measurement
☐	☒	A statement on whether measurements were taken from distinct samples or whether the same sample was measured repeatedly
☐	☒	The statistical test(s) used AND whether they are one- or two-sided<br>*Only common tests should be described solely by name; describe more complex techniques in the Methods section.*
☒	☐	A description of all covariates tested
☐	☒	A description of any assumptions or corrections, such as tests of normality and adjustment for multiple comparisons
☐	☒	A full description of the statistical parameters including central tendency (e.g. means) or other basic estimates (e.g. regression coefficient) AND variation (e.g. standard deviation) or associated estimates of uncertainty (e.g. confidence intervals)
☐	☒	For null hypothesis testing, the test statistic (e.g. F, t, r) with confidence intervals, effect sizes, degrees of freedom and P value noted<br>*Give P values as exact values whenever suitable.*
☒	☐	For Bayesian analysis, information on the choice of priors and Markov chain Monte Carlo settings
☒	☐	For hierarchical and complex designs, identification of the appropriate level for tests and full reporting of outcomes
☒	☐	Estimates of effect sizes (e.g. Cohen's d, Pearson's r), indicating how they were calculated

Our web collection on statistics for biologists contains articles on many of the points above.

Software and code

Policy information about availability of computer code

Data collection	Data were collected on Zeiss Axiovision v.4.8, Zeiss Zen 2 Blue version, QX200 ddPCR machine (Bio-Rad), 5-laser FACS Aria II (BD Biosciences), Graph pad Prism v. 7, and Microsoft Excel 2016.
Data analysis	Data were analyzed using ImageJ v.1.Slf, Image-Pro Plus v.5, QuantaSoft Software (Bio-Rad), FlowJo software, Graphpad Prism v.7, and Microsoft Excel 2016.

For manuscripts utilizing custom algorithms or software that are central to the research but not yet described in published literature, software must be made available to editors and reviewers. We strongly encourage code deposition in a community repository (e.g. GitHub). See the Nature Research guidelines for submitting code & software for further information.

Data

Policy information about availability of data

All manuscripts must include a data availability statement. This statement should provide the following information, where applicable:

- Accession codes, unique identifiers, or web links for publicly available datasets
- A list of figures that have associated raw data
- A description of any restrictions on data availability

The data that support the findings of this study are available from the corresponding author upon request.

Field-specific reporting

Please select the one below that is the best fit for your research. If you are not sure, read the appropriate sections before making your selection.

[x] Life sciences [ ] Behavioural & social sciences [ ] Ecological, evolutionary & environmental sciences

For a reference copy of the document with all sections, see nature.com/documents/nr-reporting-summary-flat.pdf

Life sciences study design

All studies must disclose on these points even when the disclosure is negative.

Sample size	Samples size was determined using statistical analyses detailed in the Methods sections to achieve significance. These sizes were based on prior experience with standard deviation and power calculations that determined the appropriate sampling number.
Data exclusions	No data was excluded from this study
Replication	All experiments were successfully replicated with biological replicate experiments.
Randomization	Islets from at least two independent donors were used for all experiments, cells were dispersed together for control and treated groups, and randomly allocated to each condition.
Blinding	Blinding was not feasible given that there was generally only two comparison groups per assay, the control and experimental group. In the experimental workflow, the experimental group was characterized and immediately confirmed to contain the targeted mutation or gene activation.

Reporting for specific materials, systems and methods

We require information from authors about some types of materials, experimental systems and methods used in many studies. Here, indicate whether each material, system or method listed is relevant to your study. If you are not sure if a list item applies to your research, read the appropriate section before selecting a response.

Materials & experimental systems

n/a	Involved in the study	
[ ]	[x]	Antibodies
[x]	[ ]	Eukaryotic cell lines
[x]	[ ]	Palaeontology and archaeology
[ ]	[x]	Animals and other organisms
[x]	[ ]	Human research participants
[x]	[ ]	Clinical data
[x]	[ ]	Dual use research of concern

Methods

n/a	Involved in the study	
[x]	[ ]	ChIP-seq
[ ]	[x]	Flow cytometry
[x]	[ ]	MRI-based neuroimaging

Antibodies

Antibodies used	goat anti-PDX1 (06-1385, Sigma-Aldrich); gp anti-INSULIN (a0564; Dako); mouse anti-glucagon (G2654 Sigma-Aldrich); Alexa Fluor 555 Goat anti Guinea Pig IgG (H+L) Secondary Antibody (A21435; Invitrogen); Alexa Fluor® 647-AffiniPure Donkey Anti-Mouse IgG (H+L) (715-605-150; Jackson ImmunoResearch)
Validation	All antibodies are commercially available and validated by respective companies. Validation with human pancreatic tissue sections was previously published by our lab.

Animals and other organisms

Policy information about studies involving animals; ARRIVE guidelines recommended for reporting animal research

Laboratory animals	NOD scid IL2Rγnull mice (stock number 005557; The Jackson Laboratory)
Wild animals	This study did not involve wild animals.
Field-collected samples	This study did not involve field-collected samples.
Ethics oversight	Animal experiments were approved by and performed in accordance with the guidelines provided by the Stanford University Institutional Animal Care and Use Committee (IACUC).

Note that full information on the approval of the study protocol must also be provided in the manuscript.

Flow Cytometry

Plots

Confirm that:

- [x] The axis labels state the marker and fluorochrome used (e.g. CD4-FITC).
- [x] The axis scales are clearly visible. Include numbers along axes only for bottom left plot of group (a 'group' is an analysis of identical markers).
- [x] All plots are contour plots with outliers or pseudocolor plots.
- [x] A numerical value for number of cells or percentage (with statistics) is provided.

Methodology

Sample preparation	Pseudoislets were dispersed into single cells by brief enzymatic digestion (Accumax, Invitrogen). Cells were stained with LIVE/DEAD™ Fixable Near-IR Dead Cell Stain Kit (Life Technologies). Non-GFP cells were used as control.
Instrument	5-laser FACS Aria II (BD Biosciences)
Software	Cytometry data was analyzed and graphed using FlowJo software (TreeStar v.10.8).
Cell population abundance	GFP+ live cells were sorted. This population was ~40%, as shown in Supplemental Figure 1.
Gating strategy	Gating controls: unstained for LIVE/DEAD™ Fixable Near-IR Dead Cell Stain Kit (Life Technologies); GFP negative were used to gate LIVE+, GFP+ populations.

- [x] Tick this box to confirm that a figure exemplifying the gating strategy is provided in the Supplementary Information.
